# Temperate versus virulent phage lifestyle impacts microdiversity in soil environments

**DOI:** 10.1093/ismeco/ycag265

**Published:** 2026-09-17

**Authors:** Thomas E P de Bruijn, Hilje M Doekes, Anne Kupczok

**Affiliations:** Bioinformatics Group, Wageningen University and Research, Droevendaalsesteeg 1, 6708PB Wageningen, The Netherlands; Laboratory of Genetics, Wageningen University and Research, Droevendaalsesteeg 1, 6708PB Wageningen, The Netherlands; Laboratory of Genetics, Wageningen University and Research, Droevendaalsesteeg 1, 6708PB Wageningen, The Netherlands; Bioinformatics Group, Wageningen University and Research, Droevendaalsesteeg 1, 6708PB Wageningen, The Netherlands

**Keywords:** bacteriophages, microdiversity, lysogeny, metagenomics, soil virome

## Abstract

Bacteriophages are not only the most ubiquitous biological entity on earth, but they also display remarkable genetic diversity across and within populations. While macrodiversity has been extensively studied, the drivers of microdiversity (intra-species genetic diversity) remain poorly understood, particularly in relation to phage lifestyle. The distinguishing ability of temperate phages to integrate themselves into the host genome has an unknown influence on the microdiversity present. This difference in microdiversity could impact the adaptability of phages to (a)biotic factors. To identify a possible association between microdiversity and lifestyle, we analysed 12 existing viromics datasets focusing on soil bacteriophages, including 43 171 viral genomes in total. We found that phages predicted to be temperate consistently exhibit higher microdiversity than their virulent counterparts in 8 of 12 datasets. Of the remaining four datasets, one showed the opposite trend and three datasets did not show a significant trend at all. The detected pattern holds across multiple quality thresholds and lifestyle prediction methods. These findings suggest that lifestyle has a significant impact on genetic variation within phage populations, with potential implications for phage–host coevolution and environmental adaptability.

## Graphical abstract

**Graphical abstract:**
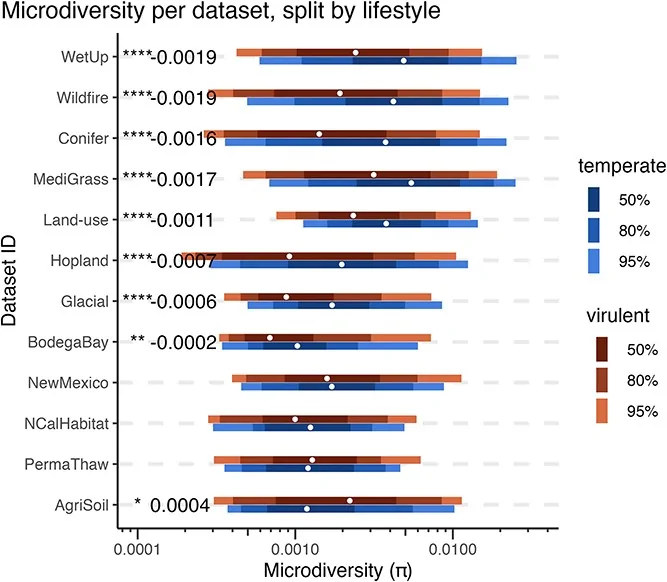

## Introduction

Viruses are known to be the most ubiquitous biological entities on Earth, exerting immense influence over prokaryotic and eukaryotic life. Bacteriophages for example shunt and shuttle nutrients throughout ecosystems by lysing up to 40% of marine bacterial populations each day [1–4]. The ubiquity of bacteriophages is reflected in—and perhaps due to—their morphological and genetic diversity [5, 6]. Advances in sequencing methods have enabled researchers to study this genetic diversity of bacteriophages at an unprecedented scale and precision [1].

Recent research into microbiome biodiversity has unearthed the vast diversity of bacteriophages, especially in soil environments [2, 7–9]. Historically, this biodiversity is described as macrodiversity or any of the many related terms (e.g. species richness, $\alpha /\beta /\gamma $-diversity) [10]. The decreased costs and improved accuracy of DNA sequencing have also paved the way to study another type of biodiversity, the so-called microdiversity. Whereas macrodiversity describes between-species diversity, microdiversity is the genetic diversity *within* a species or population.

The role microdiversity plays in the eco-evolutionary dynamics of phages cannot be uncoupled from those dynamics of the host system [11–13]. Bacteriophages and bacteria are involved in a complex coevolutionary arms race between infecting and preventing infection. Microdiversity, both in viruses and their hosts, is essential in maintaining the possibility for long-term host–virus coexistence [14]. Besides host–virus interactions, microdiversity is also necessary to provide ample variation for adaptation upon changing environmental conditions [15, 16]. Microdiversity in marine phages has been shown to be influenced by host availability, environmental stability, and virus population dynamics [1, 17]. Unfortunately, despite its importance, microdiversity in bacteriophages is still understudied compared with microdiversity in bacteria [5].

The interplay between host and phage gains an extra dimension when the lysogenic lifestyle of temperate phages is considered in contrast to the lytic lifestyle of virulent (or obligatory lytic) phages [18, 19]. Both virulent and temperate phages can perform the lytic lifecycle: the phage infects the cell, starts replication of its genome and production of necessary structural proteins, and finally induces cell lysis to release new virions. Genome replication during the lytic life cycle is affected by the viral mutation rate, which is estimated to be up to two orders of magnitude higher than the mutation rate of the host [20–22]. The lysogenic lifecycle of temperate phages involves integration of the viral genome into the host genome, instead of immediate production of virions and lysis. This turns the phage into a prophage and the host into a lysogen. The prophage can multiply with the lysogen when the cell divides, or excise itself from the host genome and return to the lytic lifestyle. Depending on biotic and abiotic factors, temperate and virulent phages can be under very different selection pressures. For example, some bacteria can transform into endospores, potentially protecting the prophage from extreme environmental conditions [23]. Furthermore, prophages can exchange genetic material with superinfecting phages or other prophages [11, 24]. However, how the lifestyle of bacteriophages affects their genetic microdiversity is currently unknown.

The ratio of phage lifestyles differs by environment, but even within environments factors like depth can make a difference [17, 25]. Overall estimates indicate a higher degree of lysogeny in soil environments versus marine environments. Besides this lifestyle ratio, soil viruses are incredibly diverse, both in structure and diversity, while also having a high potential impact on soil health through nutrient cycling and microbiome composition [26–28].

Here we studied the association between phage lifestyle and microdiversity in *Caudoviricetes*, a well-studied class of double-stranded DNA viruses that can have either a virulent or temperate lifestyle [6]. Twelve soil-borne bacteriophage datasets, selected for their recent publication and FAIR data (i.e. data were Findable, Accessible, Interoperable, and Reusable), were re-analysed. The 12 datasets together contained 537 397 viral operational taxonomic units (vOTUs, approximately species rank viral genomes), which were filtered and analysed according to best current practices [29] using a high-throughput analysis pipeline. Finally, the association between lifestyle (temperate or virulent) and microdiversity was tested on 43 171 vOTUs.

## Materials and methods

### Data collection

Datasets were selected from metagenome studies of soil-based bacteriophages catalogued in the NCBI PubMed database. Additional requirements were recent publishing (<5 years, from December 2025) and data availability of raw reads and vOTUs. Using these criteria, 17 studies were selected for further analysis (Table 1). The vOTUs and raw reads were retrieved for these datasets. vOTUs were downloaded from their respective data repositories. Raw reads were retrieved from SRA by their BioProject ID (Table 1 for IDs) using the NCBI SRA-toolkit (github.com/ncbi/sra-tools) [30]. There were slight differences how the original studies computed the vOTUs from assembled contigs. Fourteen studies followed the predominant standard for clustering of contigs into vOTUs: at least 95% average nucleotide identity over 85% alignment fraction [1, 31], whereas three studies used different parameters. Given the vOTUs from the original studies were used and no *de novo* assembly and phage identification was performed, duplicates may exist between datasets. Hence, all datasets were also re-clustered together using PSI-CD-HIT (v4.8.1, using the flags for alignment thresholds ‘-c 0.95 -G 1 -g 1 -aS 0.85 -aL 0.85”) and the standard definition for vOTUs (at least 95% average nucleotide identity over 85% alignment fraction) to check for redundancy caused by different vOTU clustering definitions between datasets [32]. This re-clustering only resulted in a very small reduction in vOTUs (see Supplemental Information, ‘Re-clustering of vOTUs using standard thresholds’); thus, we continued with the original vOTUs from each dataset.

**Table 1:** Overview of the 17 datasets used in this study.

Dataset ID	Source	BioProject ID	Clustering	No. of libraries	No. of vOTUs
AgriSoil	[7]	PRJNA646773	standard	15 (v)	4065
BodegaBay	[33]	PRJNA913601	standard	63 (v)	12 689
Conifer	[34]	PRJNA1113186	standard	90 (v)	272 017
Glacial	[9]	PRJNA882246	$\ge 95\%$ ANI over $\ge 80\%$ AF	24 (v)	59 685
Hopland	[35]	PRJNA818793	standard	44 (v)	6088
Intertidal	[36]	PRJNA957716	standard	96 (m)	20 102
Land-use	[37]	PRJNA691683	standard	50 (v)	61 106
MediGrass	[38]	PRJNA1264504	standard	59 (v)	22 725
NCalHabitat	[39]	PRJNA831438	standard	30 (v)	3432
NewMexico	[40]	PRJNA1263537	standard	11 (v)	3233
PermaThaw	[41]	PRJNA888099	standard	20 (v)	9963
Rhizo	[42]	PRJNA935076	$\ge 95\%$ ANI over $\ge 80\%$ AF	12 (v)	13 335
Spruce	[43]	PRJNA666221	standard	10 (v)	5006
UKagri	[44]	PRJEB49313	$\ge 95\%$ ANI over $\ge 95\%$ AF	15 (v)	1059
Watershed	[45]	PRJNA1168581	standard	48 (m)	2743
WetUp	[46]	PRJNA859194	standard	152 (v)	12 259
Wildfire	[47]	PRJNA1147726	standard	39 (v)	70 135

All datasets are of soil-based viruses, most of them focusing specifically on bacteriophages. Studies that performed size fractioning (*<0.22 μ *m) in their sample treatment show the number of viromes (v) or metagenomes (m). If a study had both viromes and metagenomes available, only the number of viromes is shown. Standard clustering parameters are $\ge 95\%$ ANI over $\ge 85\%$ AF. ANI, average nucleotide identity; AF, alignment fraction.

### Viral operational taxonomic unit quality assessment and mapping of reads

The files containing the vOTUs (for each dataset) were used as supplied by the authors of the respective publications. All vOTUs were analysed with CheckV (v1.0.3, ‘end_to_end’ pipeline, database version 1.5) and filtered on at least 80% estimated genome completeness [48].

Raw reads of each library were mapped against the vOTUs of the respective study with Bowtie2 (v2.4.4), using the ‘very-sensitive’ setting [49]. Unmapped reads were discarded during the mapping process, using the Bowtie2 ‘–no-unal’ flag. Bowtie2 SAM output files were converted to BAM files, sorted using SAMtools (v1.21) and merged within each dataset [50]. Coverage of contigs by mapped reads was calculated using CoverM (v0.7.0, using the contig option, ‘mean’ coverage calculation method) [51]. vOTUs were filtered for a minimum coverage of 10*× * and breadth of coverage of at least 80%. These thresholds were necessary for downstream microdiversity analysis, primarily ensuring accurate (high coverage) microdiversity estimation of the full genome (high breadth). All vOTUs were also assigned taxonomic information using geNomad (v1.11.1, default settings) [52] and filtered for being classified as *Caudoviricetes*, as the lifestyle prediction tools were designed for *Caudoviricetes* viruses [53, 54].

### Prediction of viral operational taxonomic unit lifestyle

The lifestyle of all vOTUs was predicted using PhaTYP (as included in PhaBOX2 v2.1.11) [53], PhaStyle (v0.1) [54] and verified by checking for integrases with DIAMOND (v2.0.8) [55]. PhaStyle uses a ProkBERT genomic language model to detect similarity of query sequences to phages with known lifestyles. It uses a binary classification model: the negative class in the model is the temperate lifestyle, the positive being virulent. PhaStyle was run using a GPU, greatly accelerating the processing speed compared with CPU-based usage. PhaStyle was run using the ‘neuralbioinfo/PhaStyle-mini’ model available online (huggingface.co/neuralbioinfo/prokbert-mini). PhaTYP also uses a BERT-based model, but differs from PhaStyle in the method of embedding and masking sequences [54, 56]. These tools do not identify other lifestyles, such as pseudolysogeny or chronic phages.

vOTUs were also checked for the presence of lysogeny marker genes using the cluster of orthologous genes COG0582, COG2915, COG3311, COG4570, and COG4974 from the COG database and DIAMOND (sensitive preset, e-value <1e-20 and minimum query coverage per High-Scoring Pair of 80%) [55, 57]. These COGs were chosen for their role in the lysogenic lifecycle, specifically integration (COG0582 and COG4974) and lysogen/prophage regulation (COG2915, COG3311, and COG4570). The COGs together consisted of 53 451 protein sequences and formed the database against which the proteins from all vOTUs, as predicted by Prodigal, were queried [58]. The final lifestyle classification was determined by the overlap between PhaTYP and PhaStyle and the presence of a DIAMOND hit with a lysogeny marker protein sequence for the temperate lifestyle or the absence of such a hit for the virulent lifestyle.

### Microdiversity analysis of filtered viral operational taxonomic units

All vOTUs with mapped reads were used as input for calculating microdiversity using inStrain (v1.9.1) [59]. inStrain applies a 95% ANI filter for each aligned reads, reducing spurious mappings. Default parameters were used for variant calling within inStrain (SNP coverage *≥ 5*, SNP frequency *>5%*, FDR = 1e-06). The main output metric of interest was the nucleotide diversity (*π *) per vOTU, included in the scaffold output files. Additionally, pN/pS ratios in predicted gene sequences were computed by inStrain and retrieved from the gene output files. pN/pS is the ratio between nonsynonymous and synonymous mutations in gene coding sequences and can be an indication of selective pressure on a genetic sequence. For datasets with at least 10 vOTUs per lifestyle, the ratio between single library microdiversity and global microdiversity (i.e. the per-dataset approach) was also calculated.

### Association analysis of lifestyle–microdiversity relationship

The association analysis between phage lifestyle and microdiversity was performed using R (v4.4.2) [60] and additional libraries from the tidyverse were used for data handling and visualization [61]. We required there to be at least 10 vOTUs in each lifestyle group per dataset analysed. A Wilcoxon rank-sum test was used to calculate the statistical relationship between the two lifestyle groups and their respective microdiversity.

### Functional annotation of viral genes

vOTUs belonging to the Land-use dataset also underwent functional prediction of genes. For this, genes were first predicted using Prodigal (v2.6.3, anonymous mode) [58]. After this, eggNOG mapper (v2.1.13, default settings) was used in combination with the eggNOG v5 database to predict the function of genes in vOTUs [62, 63]. Annotations were parsed for keywords in the GFF fields for predicted PFAM domains (em_PFAMS) and description (em_desc). Since the focus was on essential genes, only the genes containing the following values in the GFF fields ‘em_desc’ and ‘em_PFAMs’ were highlighted during visualization: ‘terminase’, ‘capsid / head / coat’, ‘portal’, ‘tail’, ‘helicase’, ‘integrase’, and ‘recombinase’.

### SRR2strain

The workflow presented here has been largely integrated into a single Snakemake pipeline (v9.3.3), called SRR2strain (https://github.com/thattimetho/SRR2strain, Supplemental Fig. 1). Snakemake is a python-based workflow management system that allows the linking of input and output of tools to automate workflows [64]. The steps mentioned here, from the retrieval of the raw reads up until the microdiversity analysis using inStrain, have been incorporated into the pipeline. The acquisition of the respective vOTU databases, functional annotation of Land-use vOTUs, and final analysis using R scripts are not incorporated in the pipeline.

## Results

### Viromes facilitate stringent filtering required for reliable microdiversity estimation

A systematic literature search was performed for studies on bacteriophages and soil environments, taking into account the recency of publication and availability of data. This search resulted in 17 datasets. These 17 datasets formed the starting point of our analysis into the relationship between phage lifestyle and microdiversity. Of the 17 datasets, two contained only metagenomes, while the rest contained only viromes or both viromes and metagenomes (Table 1). The datasets are from diverse soil environments (e.g. coastal, agricultural, and urban). These datasets were used as input for the SRR2strain pipeline (Supplemental Fig. 1, also see subsection SRR2strain), which predicts phage lifestyle and estimates phage microdiversity. As part of this pipeline, sequenced reads are mapped back onto the vOTUs to allow for the calculation of microdiversity downstream (using inStrain) [59]. During this mapping process, we observed that the metagenomes showed extremely poor mapping efficiency (<3% mapped reads, Supplemental Table 1). This was irreconcilable with the thresholds on minimum mapping breadth and coverage necessary for accurate microdiversity estimation. For this reason, the samples with only metagenomes (Watershed and Intertidal) were excluded from the analysis. Only viromes were considered for the remaining datasets and analyses, which led to a significant improvement in mapping efficiency (see Supplemental Table 1).

The 15 remaining datasets were filtered for sufficient mapping coverage and breadth, and vOTU completeness. This led to the removal of Rhizo, Spruce, and UKagri, as these datasets did not have sufficient vOTUs (at least 10 vOTUs per lifestyle, see Materials and methods) left for lifestyle microdiversity association analysis. From the other 12 datasets, 117 022 out of 537 397 vOTUs remained after these (mapping) quality control measures were applied. Subsequent taxonomic classification was performed using geNomad and resulted in successful classification of 116 445 vOTUs (99.5% of 117 022 total). Of these, 115 922 (99.6%) were predicted to belong to the *Caudoviricetes* class of viruses. Of the remaining non *Caudoviricetes* 523 vOTUs, 411 were assigned as *Tectiviridae*, 83 as *Priklausovirales*, with the remainder spread across various *Monodnaviria* and *Varidnaviria* subtaxa. For subsequent analyses and results, only the vOTUs classified as belonging to the *Caudoviricetes* class were considered.

### Virulent phages comprise majority in lifestyle distribution

Phage lifestyle, either virulent or temperate, was predicted for the 115 922 *Caudoviricetes* vOTUs after quality filtering using a combination of tools (PhaTYP, PhaStyle, and DIAMOND) (Table 2). This resulted in 43 171 vOTUs which were assigned the same lifestyle by both PhaTYP and PhaStyle, requiring temperate phages to have a lysogeny marker protein detected by DIAMOND. Of these 43 171 vOTUs, 11 524 (27%) were classified as having a temperate lifestyle and 31 647 (73%) as having a virulent lifestyle. The ratio between virulent and temperate phages per study was similar between all 12 studies (Fig. 1). The lifestyle ratio changed slightly when corrected for relative abundance of vOTUs, leading to temperate phages increasing in proportion (Supplemental Fig. 2). In total, 72 751 vOTUs that passed the initial stringent quality checks were discarded due to disagreement between lifestyle prediction methods (Fig. 1A).

**Table 2:** Overview of datasets used as input for microdiversity analysis.

Dataset ID	No. of vOTUs	No. of vOTUs	No. of vOTUs	No. of vOTUs	No. of vOTUs
	total	quality filtered	quality and lifestyle filtered	predicted temperate	predicted virulent
AgriSoil	4065	725	224	85	139
BodegaBay	12 689	904	264	65	199
Conifer	272 017	58 095	22 335	5281	17 054
Glacial	59 685	15 051	6517	1381	5136
Hopland	6088	859	280	94	186
Intertidal	20 102	–	–	–	–
Land-use	61 106	13 865	4543	1440	3103
MediGrass	22 725	10 631	3557	1003	2554
NCalHabitat	3432	373	121	32	89
NewMexico	3233	1159	399	119	280
PermaThaw	9963	660	245	53	192
Rhizo	13 335	–	–	–	–
Spruce	5006	–	–	–	–
UKagri	1059	–	–	–	–
Watershed	2743	–	–	–	–
WetUp	12 259	5107	1764	862	902
Wildfire	70 135	8493	2922	1109	1813

The total column indicates input data, whereas the quality filtered column indicates quality and taxonomy filtered data. Taxonomic classification resulted in almost all quality filtered vOTUs being classified as *Caudoviricetes* viruses. The last two columns are based on total number of quality, taxonomy, and lifestyle filtered vOTUs, split by lifestyle. The sum of the lifestyles is shown in the ‘No. of vOTUs quality and lifestyle filtered’ column.

**Figure 1:**
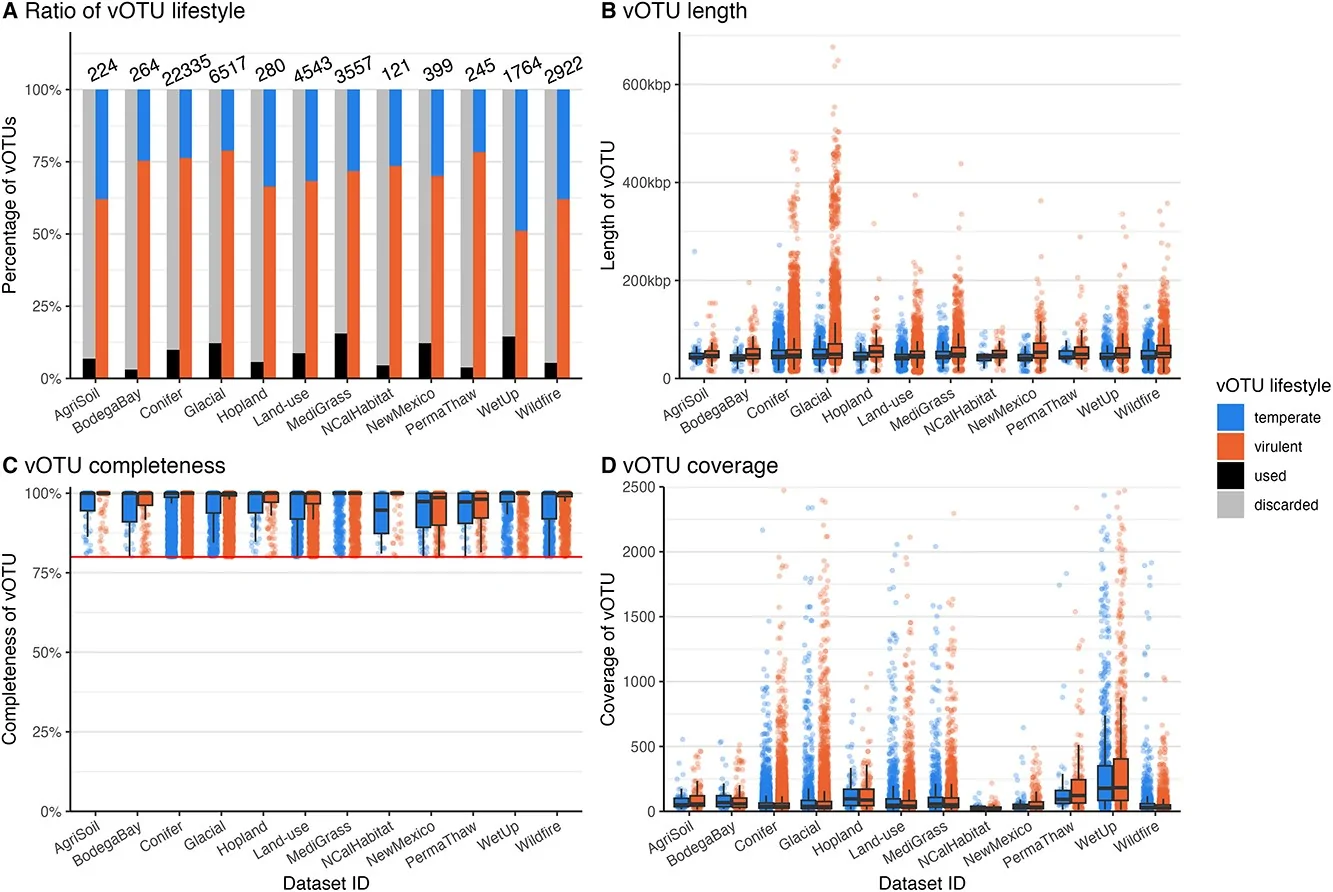
Overview of vOTU-related statistics. (A) Ratios of total and used data, and the ratio between temperate and virulent classified vOTUs. The numbers indicate total vOTUs used for microdiversity analysis. (B) Length distribution of used vOTUs among the 12 datasets. Strikingly, only the Glacial and Conifer study show multiple viral genome lengths above 400 kb. (C) Predicted completeness of used vOTUs. The used threshold of 80% is indicated with a solid horizontal line. (D) Mapping coverage of used vOTUs. The threshold for coverage was put at 10*× *.

In order to identify and assess any confounding variables, various other metrics, like mapping breadth, coverage, vOTU length and completeness, have also been tested in relation to each other and to nucleotide diversity (Spearman correlation, alpha = 0.05) (Supplemental Fig. 3). The only consistent significant correlation found by this analysis was between mapping coverage and breadth. Some inconsistent correlations have also been found, like those between microdiversity and genome length, and microdiversity and pN/pS. The association of lifestyle with the aforementioned variables has also been tested (Wilcoxon rank-sum test, two-tailed). Some of these metrics show a significant statistical association with vOTU lifestyle, but the estimated true difference between the groups is very small (Fig. 1B and C and Supplemental Table 2).

### Overview of high-quality viral datasets

After filtering on mapping and vOTU quality and taking congruent lifestyle predictions, the final assembled dataset consisted of 12 datasets, containing 43 171 vOTUs (8% of all 537 397 vOTUs in these datasets, see also Table 2). The included vOTUs were not distributed equally among the 12 studies: *∼ *50% originated from the Conifer dataset. On the dataset level, average mapping breadth and estimated vOTU completeness showed similar values among the 12 studies, while average vOTU length and average coverage showed more variability between datasets (Fig. 1 and Supplemental Tables 3 and 4). Median mapping coverage showed values between 21*× * (NCalHabitat) and 186*× * (WetUp).

When split by lifestyle, correlation analysis (Spearman correlation, alpha = 0.05, see Supplemental Fig. 3 and also Table 4) indicated a general correlation between microdiversity and length, with 8 out of 12 datasets showing a negative correlation. This was also supported by an association analysis (Wilcoxon rank-sum test, two-tailed, see Supplemental Table 2). Since the estimated effect is small and microdiversity is corrected for genome length, no additional corrections were applied as a result of this.

### Soil-borne bacteriophage microdiversity shows association with lifestyle

Microdiversity (*π *) present within the vOTUs was calculated to identify and analyse any association with phage lifestyle. Generally, microdiversity levels were similar between studies and consistent with the microdiversity reported in the original studies [9, 37]. Overall, the studies showed quite a wide range of microdiversity (Fig. 2A), ranging from a median of 0.0010 (BodegaBay) to a median of 0.0054 (MediGrass). When comparing single libraries to the global (pooled) dataset, microdiversity per library is generally more than half of the dataset microdiversity (see Supplemental Fig. 4).

**Figure 2:**
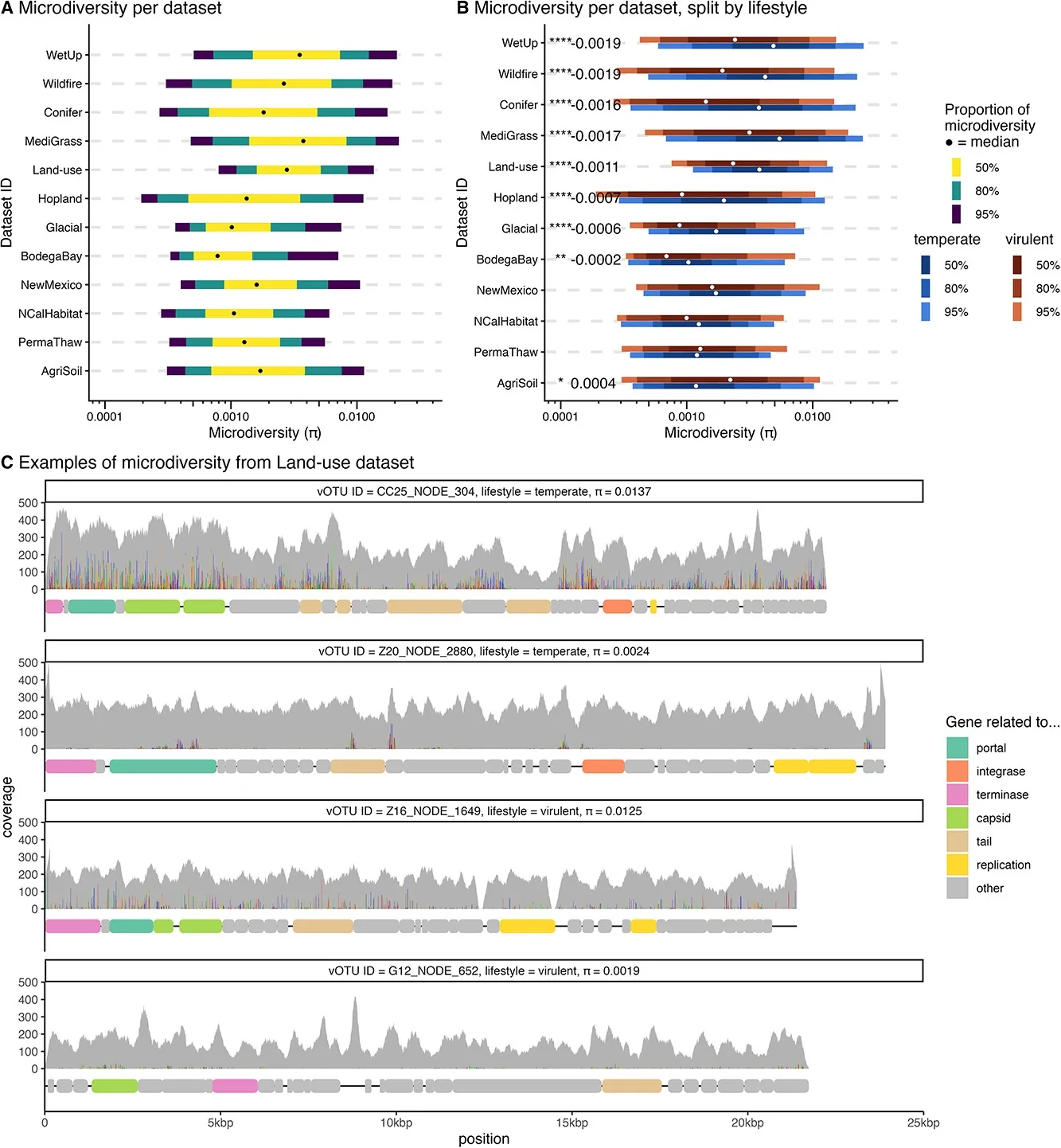
Distribution of microdiversity in temperate phages and virulent phages across 12 datasets. (A and B) Datasets are sorted by difference in microdiversity between lifestyles. (A) Interval plot of microdiversity distribution of vOTUs. (B) Interval plot of microdiversity distribution split by lifestyle indicating significance (^*^^*^^*^^*^P<0.0001, ^*^^*^^*^P<0.001, **P<0.01, ^*^P<0.05, Wilcoxon rank sum test, two sided) and value next to significance indication shows estimated difference between distributions. Higher microdiversity in temperate phages is shown by the more right skewed microdiversity distributions of the temperate phages. (C) Four Land-use vOTUs showing different microdiversity and lifestyle. Of each lifestyle, both a high and low microdiversity example is given. Coverage for the dataset (read identity >95%) and gene annotation across the genomes are shown.

We find that 9 out of 12 datasets showed a significant difference in the level of microdiversity detected for each lifestyle (Fig. 2B and Supplemental Table 5). Only one of these (AgriSoil) shows a higher level of microdiversity in virulent phage. In contrast, the vast majority of the significant datasets showed a higher level of microdiversity in temperate phages compared with virulent phages, where the WetUp dataset showed the most distinct microdiversity distributions between the two lifestyles (*P = 6.4*e-36, estimate = 0.0019, Wilcoxon rank-sum test, two-tailed). The Conifer dataset contained the most vOTUs out of all datasets, which influenced the statistical significance of the difference in microdiversity between the lifestyles (*P = 0*, estimate = 0.0016, Wilcoxon rank-sum test, two-tailed). Overall, the association between lifestyle and microdiversity was robust when using different lifestyle prediction methods and when correcting for skewed lifestyle ratios (see Supplemental Table 5 and Fig. 5).

When inspecting read mapping across example genomes, we find reasonable coverage patterns, containing no clearly depleted or significantly enriched areas of the genome (Fig. 2C). The coverage of the vOTUs also does not seem to correlate strongly with the amount of microdiversity, which is supported by the general correlation analysis on the Land-use dataset (Spearman correlation, alpha = 0.05, Supplemental Fig. 3). The localization of microdiversity seems to vary by vOTU, with Z20_NODE_2880 seemingly showing more microdiversity in the terminase and portal protein predicted proteins compared with the rest of the genome. Meanwhile, Z16_NODE_1649 shows a more even distribution of microdiversity throughout the vOTU.

In addition to vOTU-wide microdiversity analysis, the effect of lifestyle on gene-level characteristics was also studied. Besides general statistics, like length and breadth (see Supplemental Fig. 6),

To analyse the association of selective pressure on genes with lifestyle, we analysed the pN/pS ratios as calculated by inStrain. Generally, ratios between 0 and 1 indicate purifying selection, ratios around 1 are a sign of neutral protein evolution and ratios above 1 indicate positive selection. We find that the majority of pN/pS ratios is below 1, indicating purifying selection on the vOTUs (Fig. 3). Phage lifestyle showed a significant association with pN/pS in 11 out of 12 datasets (Wilcoxon rank sum test, see also Supplemental Table 6), where temperate phages showed higher pN/pS than virulent phages. Lastly, correlation analysis (Spearman correlation, alpha = 0.05) shows a strong positive correlation between the pN/pS ratio and microdiversity in 10 out of 12 datasets (Supplemental Figs 3 and 4).

**Figure 3:**
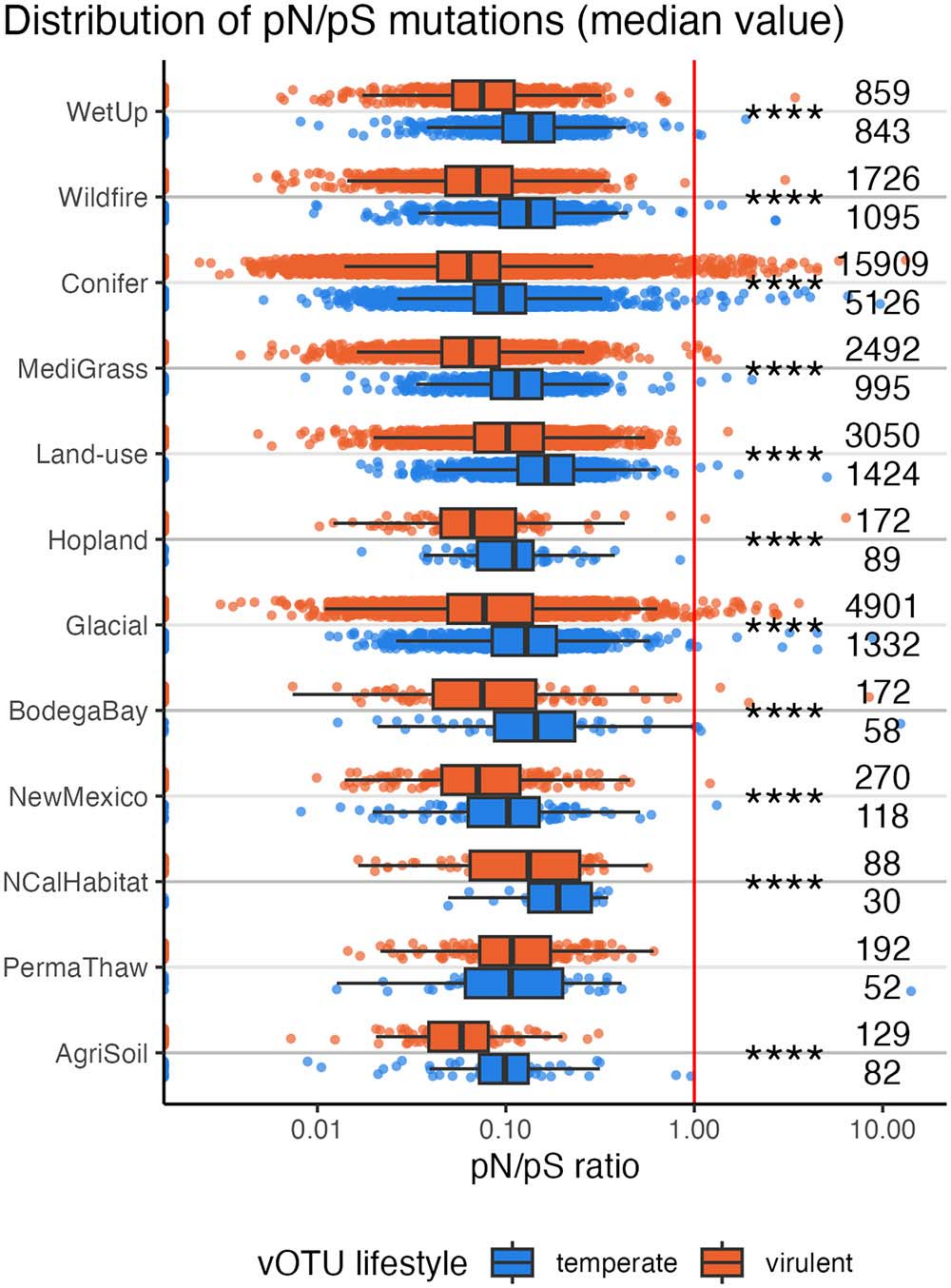
pN/pS ratios per dataset split by lifestyle. Temperate phages show consistently and significantly higher pN/pS ratios than virulent phages. Difference in pN/pS between lifestyle was statistically tested with a Wilcoxon rank sum test, resulting in significant differences in the pN/pS values of 11 of 12 datasets between temperate and virulent lifestyles as indicated by the asterisks (see Supplemental Table 6). The numbers on the right indicate the number of vOTUs that were included in the Wilcoxon rank sum test and plot.

## Discussion

Advances in high-throughput, cost-effective sequencing and improved assembly have led to an expanding set of high-quality DNA viromic datasets from diverse environments, including soils. Leveraging these resources, we find that phage lifestyle is associated with microdiversity across different soil datasets. Using stringent filters for both read mapping and lifestyle prediction, we obtained confident association statistics for 12 datasets; 9 of the 12 showed significant lifestyle-associated differences, with 8 showing temperate phages displaying higher microdiversity (Fig. 2). Across those eight datasets, statistics show an estimated *∼ *36% difference in microdiversity between the two lifestyles. Overall microdiversity levels and lifestyle distribution were in line with those reported by previous soil-focused studies [9, 36, 37]. Furthermore, the pN/pS ratio indicates of the type of selection pressure on protein-coding genes. We find generally low pN/pS ratios, indicating that the vOTUs are under purifying selective pressure and pN/pS rations are generally lower for virulent phages (Fig. 3). Purifying selection can be a sign of a relatively unperturbed environment, thus not favouring new variants or gene functions to allow for new niche occupation [65]. However, soil is usually seen as a very dynamic environment, especially topsoil, which is generally sampled [43, 66]. Notably, genome-wide purifying selection might hide the signatures of positive selection on only a few protein regions. Additional analyses are necessary to investigate how the distribution of these mostly synonymous mutations is spread across the gene repertoire of phages and whether these mutations localize in certain groups of genes.

As this project aimed to connect microdiversity with phage lifestyle, accurate microdiversity estimation is key. Microdiversity analysis requires a high read depth, as to filter out sequencing errors and provide certainty on the amount of genetic diversity present. Our initial collection of datasets contained two studies with metagenomes only rather than (size-fractionated) viromes. However, mapping the metagenomic reads against the vOTUs resulted in low mapping efficiency and low coverages of vOTUs and consequently to a low number of vOTUs with sufficient coverage. This is consistent with previous reports that metagenomes do not contain viral reads at sufficient coverage for downstream analysis [7, 67]. Soil samples are especially hard to sequence for viruses using metagenomics, underlining the need for viromes further [67, 68]. Even when using viromes, the strict quality thresholds we imposed substantially reduced analysable vOTUs. For example, in the BodegaBay dataset, this led to a reduction in vOTUs of 98%, of which: 64% did not pass the quality thresholds (but could be assigned a lifestyle with certainty), and 34% lacked confident lifestyle assignment. For three datasets (Rhizo, Spruce, and UKagri) the stringent filtering resulted in too few vOTUs for association analysis, which could be due to the low number of libraries and total reads in these datasets (Table 1and Supplemental Table 1). This results in a low overall coverage and highlights the importance of deeply-sequenced viromes for such fine-grained analyses. The pooling of viromes, as done in this study, is a way to further increase the number of vOTUs with high coverage and breadth, but does mean the analysis and results should be considered to be on an environmental level, not a local one. This is also seen when comparing per-sample microdiversity and pooled microdiversity, with all datasets showing a higher pooled microdiversity (Supplemental Fig. 7).

Not only the data used influences the output achieved, so do the various clustering and mapping methods, tool settings and filtering steps. For example, the pipeline only includes a single mapping tool, Bowtie2. Different mappers with different settings could influence the mapping efficiency and accuracy. Nevertheless, we expect only a limited effect of using another mapping tool, given that the reads from all datasets were mapped with the same tool and the mapping is independent of phage lifestyle. The subsequent filtering using both coverage and breadth was aimed at reducing spurious mappings. In addition to these thresholds, inStrain was run using default filtering steps, which also includes an ANI cutoff of 95% of mapped reads. Overall, given the many strict thresholds set on vOTU and mapping quality, we are confident that the results presented here are a realistic representation of the microdiversity present in the respective datasets.

In this study, bacteriophage lifestyle is hypothesized to be an explaining variable of microdiversity, and as such, lifestyle prediction needs to be as accurate and precise as possible. Because of this, we combined two sequence-based lifestyle prediction tools, namely PhaTYP and PhaStyle, with DIAMOND search against lysogeny marker genes [54–56]. In addition to choosing the overlap between these three tools, various combinations of tools have also been tested, which shows that the microdiversity results are robust towards the chosen lifestyle prediction tool (Supplemental Table 5). PhaTYP and PhaStyle were chosen for their recent development and extensive benchmarking against established but older tools [54, 56]. Despite similar conceptual underpinnings, PhaTYP and PhaStyle yielded substantial variance in lifestyle calls (Supplemental Fig. 8). This subsequently led to 72 751 vOTUs being filtered out due to disagreement between the lifestyle prediction results (Table 2). This difference between tools is the exact reason we combine the predictions of multiple tools. It also highlights the difficulty of accurate lifestyle prediction. The actual ratio between virulent and temperate phages in soil may differ from our current estimates from the filtered datasets, either due to lifestyle prediction methods or sampling bias. To improve lifestyle prediction *a posteriori* for studies containing both metagenomes and viromes, assembled bacterial genomes could be checked for prophages also present in the viral fraction, thus indicating active lysogeny. Other options of integrating orthogonal evidence to reduce uncertainty in lifestyle assignment include experimental methods like studying the inducible fraction or bioinformatic methods like prophage signature analysis or long-read phage–host linkage [25, 26, 69, 70].

Our results indicate a consistent difference in pN/pS ratio between lifestyles and indicate an impact of lifestyle on microdiversity. In order to explain these differences, we can take a look at the various evolutionary factors that influence viral populations. Selective sweeps and mutation rates are expected to contribute to microdiversity particularly in large, rapidly reproducing viral populations. The exact link between these processes and the consistent lifestyle-dependent differences we detect is not clear. Several nonmutually exclusive hypotheses could account for elevated microdiversity in temperate phages. First, temperate phages can alternate between lysogenic and lytic replication, potentially increasing opportunities for accumulation and maintenance of within-species polymorphisms across fluctuating ecological conditions and host states [23, 71–73]. These fluctuating ecological conditions are expected to lead to strong population bottlenecks, especially for virulent phages, as those do not have the option to integrate themselves into a host besides pseudolysogeny. Pseudolysogeny is a stalled state of a phage infection, and could mean virulent phages behave similar to the prophage state of temperate phages [74]. Nonetheless, the harsh world that phages live in is expected to lead to hard selective sweeps, where a genome carrying advantageous mutations increases in frequency leading to a substantial reduction in diversity [75]. Due to large phage population sizes, we expect even a minor advantageous mutation to rapidly fix in this environment. These hard selective sweeps may remove a part of the microdiversity from the virulent phage population, but not *per se* from temperate phages which are integrated into a host. This could explain both pN/pS lifestyle association, as well as the correlation between microdiversity and pN/pS, either by temperate phages ‘hiding’ from natural selection, or by virulent phages losing diversity through background selection and population bottlenecks [19]. Second, integration into diverse host genomes may expose temperate phages to host-mediated selection and recombination landscapes, which can elevate microdiversity relative to strictly lytic counterparts [76–78]. Lastly, recurrent induction events may repeatedly reseed the temperate phage population with genetically diverse lineages that have diversified due to genetic drift and mutation during prolonged periods of integration [19, 23, 79].

Soil environments are highly spatially heterogeneous, leading to microcosms containing spatially structured bacterial and bacteriophage populations. Prior work has shown that soil viral communities can harbour substantially greater microdiversity than those from marine systems, likely due to this spatial heterogeneity [1, 37, 39]. The unique spatial properties of soil might also be essential for the pronounced lifestyle signal we observe, given that spatial heterogeneity can greatly influence both phage and host population dynamics [16, 35, 39, 80, 81]. Moreover, while the initial systematic dataset search did not take into account the diversity of sampled biomes, it is worth mentioning that the identified lifestyle–microdiversity association is present across multiple different biomes, like the forests of the Conifer and Wildfire study, and the multiple grassland biomes of the Hopland and MediGrass studies [34, 35, 38, 47]. Yet, while the lifestyle–microdiversity association seems robust, there is one dataset, AgriSoil (Fig. 2), showing the opposite trend to the other eight significant associations. However, this dataset has relatively low read numbers and the bootstrapping shows less consistency than for the other significant associations (Supplemental Table 1 and Fig. 5). Thus, this difference might be caused by methodological differences to the other datasets or it could be a genuine biological result, where specific ecological factors in the environment contribute to the higher microdiversity in virulent phages or the lower microdiversity in temperate phages.

In sum, across multiple high-quality soil viromics datasets, phage lifestyle impacts microdiversity, generally leading to higher microdiversity in temperate phages than virulent phages, a pattern robust to alternative prediction tools and filtering thresholds and present throughout biomes. This suggests that lifestyle-linked ecological and evolutionary processes contribute to the association between lifestyle and microdiversity. Limited by the data and methods available, we can only hypothesize on potential explanations for this apparent association between lifestyle and microdiversity at the moment. Further investigation, taking into account the complexity of soil environments and the location of diversity across different genomic regions, is warranted to identify the ecological and evolutionary mechanisms driving the consistent lifestyle–microdiversity association.

## Supplementary Material

TdB_PhD_Chapter_1_supp_full_v3_ycag265

## Data Availability

A list of IDs and locations for raw read data and vOTU datasets as well as data generated and used for generating visualizations in this study has been uploaded to Zenodo (https://doi.org/10.5281/zenodo.17988606). Raw read data of publications used in this study are available on the NCBI SRA database. vOTU datasets of publications used in this study were retrieved from their respective online data repositories. The SRR2strain snakemake pipeline and all other scripts/code generated for this study are available on GitHub (https://github.com/thattimetho/SRR2strain). This repository also contains information on all the tools and libraries used in the processing or visualization of data.

## References

[1] Gregory AC, Zayed AA, Conceição-Neto N, et al. Marine DNA viral macro- and microdiversity from pole to pole. *Cell* 2019; 177:1109–1123.e14. 10.1016/j.cell.2019.03.040 PMC6525058 31031001

[2] Roux S, Emerson JB. Diversity in the soil virosphere: to infinity and beyond? *Trends Microbiol* 2022;30:1025–35. 10.1016/j.tim.2022.05.003 35644779

[3] Jansson JK, Wu R. Soil viral diversity, ecology and climate change. *Nat Rev Microbiol* 2023;21:296–311. 10.1038/s41579-022-00811-z 36352025

[4] Piedade GJ, Schön ME, Lood C, et al. Seasonal dynamics and diversity of Antarctic marine viruses reveal a novel viral seascape. *Nat Commun* 2024;15:9192. 10.1038/s41467-024-53317-y PMC11502894 39448562

[5] Dion MB, Oechslin F, Moineau S. Phage diversity, genomics and phylogeny. *Nat Rev Microbiol* 2020;18:125–38. 10.1038/s41579-019-0311-5 32015529

[6] Roux S, Coclet C. Viromics approaches for the study of viral diversity and ecology in microbiomes. *Nat Rev Genet* 2026;27:32–46. 10.1038/s41576-025-00871-w 40691354

[7] Santos-Medellin C, Zinke LA, Ter Horst AM, et al. Viromes outperform total metagenomes in revealing the spatiotemporal patterns of agricultural soil viral communities. *ISME J* 2021;15:1956–70. 10.1038/s41396-021-00897-y PMC8245658 33612831

[8] Chen T, Xiong Y, Zhang J, et al. Temporal dynamics, microdiversity, and ecological functions of viral communities during cyanobacterial blooms in Lake Taihu. *NPJ Biofilms Microbi* 2025;11:178. 10.1038/s41522-025-00771-1 PMC12397367 40883308

[9] Liao H, Li J, Wang Y-Z, et al. Evolutionary diversification and succession of soil huge phages in glacier foreland. *Microbiome* 2025;13:18. 10.1186/s40168-024-02017-2 PMC11748809 39838455

[10] Kimura M, Jia Z-J, Nakayama N, et al. Ecology of viruses in soils: past, present and future perspectives. *Soil Sci Plant Nutr* 2008;54:1–32. 10.1111/j.1747-0765.2007.00197.x

[11] Brüssow H, Canchaya C, Hardt W-D. Phages and the evolution of bacterial pathogens: from genomic rearrangements to lysogenic conversion. *Microbiol Mol Biol Rev* 2004;68:560–602. 10.1128/MMBR.68.3.560-602.2004 PMC515249 15353570

[12] Koskella B, Brockhurst MA. Bacteria-phage coevolution as a driver of ecological and evolutionary processes in microbial communities. *FEMS Microbiol Rev* 2014;38:916–31. 10.1111/1574-6976.12072 PMC4257071 24617569

[13] Burmeister AR, Lenski RE, Meyer JR. Host coevolution alters the adaptive landscape of a virus. *Proc Biol Sci* 2016;283:20161528. 10.1098/rspb.2016.1528 PMC5046904 27683370

[14] Piel D, Bruto M, Labreuche Y, et al. Phage-host coevolution in natural populations. *Nat Microbiol* 2022;7:1075–86. 10.1038/s41564-022-01157-1 35760840

[15] Larkin AA, Martiny AC. Microdiversity shapes the traits, niche space, and biogeography of microbial taxa. *Environ Microbiol Rep* 2017;9:55–70. 10.1111/1758-2229.12523 28185400

[16] García-García N, Tamames J, Linz AM, et al. Microdiversity ensures the maintenance of functional microbial communities under changing environmental conditions. *ISME J* 2019;13:2969–83. 10.1038/s41396-019-0487-8 PMC6864100 31417155

[17] Coutinho FH, Rosselli R, Rodríguez-Valera F. Trends of microdiversity reveal depth-dependent evolutionary strategies of viruses in the Mediterranean. *mSystems* 2019;4:e00554–19. 10.1128/mSystems.00554-19 PMC6832022 31690594

[18] Hobbs Z, Abedon ST. Diversity of phage infection types and associated terminology: the problem with lytic or lysogenic. *FEMS Microbiol Lett* 2016;363:fnw047. 10.1093/femsle/fnw047 26925588

[19] Wahl LM, Pattenden T. Prophage provide a safe haven for adaptive exploration in temperate viruses. *Genetics* 2017;206:407–16. 10.1534/genetics.116.197541 PMC5419484 28315835

[20] Sanjuán R, Nebot MR, Chirico N, et al. Viral mutation rates. *J Virol* 2010;84:9733–48. 10.1128/JVI.00694-10 PMC2937809 20660197

[21] Kupczok A, Neve H, Huang KD, et al. Rates of mutation and recombination in Siphoviridae phage genome evolution over three decades. *Mol Biol Evol* 2018;35:1147–59. 10.1093/molbev/msy027 PMC5913663 29688542

[22] Peck KM, Lauring AS. Complexities of viral mutation rates. *J Virol* 2018;92:e01031–17. 10.1128/JVI.01031-17 PMC6026756 29720522

[23] Van Goethem MW, Swenson TL, Trubl G, et al. Characteristics of wetting-induced bacteriophage blooms in biological soil crust. *mBio* 2019;10:e02287–19. 10.1128/mBio.02287-19 PMC6918073 31848272

[24] Moineau S, Pandian S, Klaenhammer TR. Evolution of a lytic bacteriophage via DNA acquisition from the *Lactococcus lactis* chromosome. *Appl Environ Microbiol* 1994;60:1832–41. 10.1128/aem.60.6.1832-1841.1994 PMC201570 16349277

[25] Williamson KE, Radosevich M, Smith DW, et al. Incidence of lysogeny within temperate and extreme soil environments. *Environ Microbiol* 2007;9:2563–74. 10.1111/j.1462-2920.2007.01374.x 17803780

[26] Liang X, Zhang Y, Wommack KE, et al. Lysogenic reproductive strategies of viral communities vary with soil depth and are correlated with bacterial diversity. *Soil Biol Biochem* 2020;144:107767. 10.1016/j.soilbio.2020.107767

[27] Brown TL, Charity OJ, Adriaenssens EM. Ecological and functional roles of bacteriophages in contrasting environments: marine, terrestrial and human gut. *Curr Opin Microbiol* 2022;70:102229. 10.1016/j.mib.2022.102229 36347213

[28] Hazard C, Anantharaman K, Hillary LS, et al. Beneath the surface: unsolved questions in soil virus ecology. *Curr Opin Microbiol* 2025;205:109780. 10.1016/j.soilbio.2025.109780

[29] Wendling CC, Vasse M, Wielgoss S. Phage quest: a beginner’s guide to explore viral diversity in the prokaryotic world. *Brief Bioinform* 2025;26:bbaf449. 10.1093/bib/bbaf449 PMC12406692 40900113

[30] Sayers EW, Bolton EE, Brister JR, et al. Database resources of the national center for biotechnology information. *Nucleic Acids Res* 2022;50:D20–6. 10.1093/nar/gkab1112 PMC8728269 34850941

[31] Roux S, Adriaenssens EM, Dutilh BE, et al. Minimum information about an uncultivated virus genome (MIUViG). *Nat Biotechnol* 2019;37:29–37. 10.1038/nbt.4306 PMC6871006 30556814

[32] Fu L, Niu B, Zhu Z, et al. CD-HIT: accelerated for clustering the next-generation sequencing data. *Bioinformatics (Oxford, England)* 2012;28:3150–2. 10.1093/bioinformatics/bts565 PMC3516142 23060610

[33] Ter Horst AM, Fudyma JD, Sones JL, et al. Dispersal, habitat filtering, and eco-evolutionary dynamics as drivers of local and global wetland viral biogeography. *ISME J* 2023;17:2079–89. 10.1038/s41396-023-01516-8 PMC10579374 37735616

[34] Geonczy SE, Hillary LS, Santos-Medellín C, et al. Patchy burn severity explains heterogeneous soil viral and prokaryotic responses to fire in a mixed conifer forest. *mSystems* 2025;10:e0174924. 10.1128/msystems.01749-24 PMC12172441 40366158

[35] Santos-Medellín C, Estera-Molina K, Yuan M, et al. Spatial turnover of soil viral populations and genotypes overlain by cohesive responses to moisture in grasslands. *Proc Natl Acad Sci USA* 2022;119:e2209132119. 10.1073/pnas.2209132119 PMC9659419 36322723

[36] Ji M, Zhou J, Li Y, et al. Biodiversity of mudflat intertidal viromes along the Chinese coasts. *Nat Commun* 2024;15:8611. 10.1038/s41467-024-52996-x PMC11452619 39367024

[37] Hu L, Hu L, Duan C-S, et al. Response of soil viral communities to land use changes. *Nat Commun* 2022;13:6027. 10.1038/s41467-022-33771-2 PMC9556555 36224209

[38] Fudyma JD, Penev P, Estera-Molina K, et al. Spatial and depth structuring predominate over temporal variation in Mediterranean grassland soil viral communities. *ISME Commun* 2026;6:ycag189. 10.1093/ismeco/ycag189 PMC13494778 42630919

[39] Durham DM, Sieradzki ET, Ter Horst AM, et al. Substantial differences in soil viral community composition within and among four northern California habitats. *ISME Commun* 2022;2:100. 10.1038/s43705-022-00171-y PMC9723544 37938790

[40] Feeser K, Longley R, Gallegos-Graves LV, et al. Recovering new viruses from New Mexico soils. *Microbiol Resour Announc* 2025;14:e0090825. 10.1128/mra.00908-25 PMC12613941 41099501

[41] Trubl G, Roux S, Borton MA, et al. Population ecology and biogeochemical implications of ssDNA and dsDNA viruses along a permafrost thaw gradient. *Nat Commun* 2025;17:375. 10.1038/s41467-025-67057-0 PMC12796364 41381546

[42] Braga LPP, Schumacher RI. Awaking the dormant virome in the rhizosphere. *Mol Ecol* 2023;32:2985–99. 10.1111/mec.16893 36807953

[43] Ter Horst AM, Santos-Medellín C, Sorensen JW, et al. Minnesota peat viromes reveal terrestrial and aquatic niche partitioning for local and global viral populations. *Microbiome* 2021;9:233. 10.1186/s40168-021-01156-0 PMC8626947 34836550

[44] Muscatt G, Hilton S, Raguideau S, et al. Crop management shapes the diversity and activity of DNA and RNA viruses in the rhizosphere. *Microbiome* 2022;10:181. 10.1186/s40168-022-01371-3 PMC9590211 36280853

[45] Coclet C, Sorensen PO, Karaoz U, et al. Virus diversity and activity is driven by snowmelt and host dynamics in a high-altitude watershed soil ecosystem. *Microbiome* 2023;11:237. 10.1186/s40168-023-01666-z PMC10604447 37891627

[46] Santos-Medellín C, Blazewicz SJ, Pett-Ridge J, et al. Viral but not bacterial community successional patterns reflect extreme turnover shortly after rewetting dry soils. *Nat Ecol Evol* 2023;7:1809–22. 10.1038/s41559-023-02207-5 37770548

[47] Geonczy SE, Ter Horst AM, Emerson JB. Soil viral communities shifted significantly after wildfire in chaparral and woodland habitats. *ISME Commun* 2025;5:ycaf073. 10.1093/ismeco/ycaf073 PMC12085916 40385308

[48] Nayfach S, Camargo AP, Schulz F, et al. CheckV assesses the quality and completeness of metagenome-assembled viral genomes. *Nat Biotechnol* 2021;39:578–85. 10.1038/s41587-020-00774-7 PMC8116208 33349699

[49] Langmead B, Salzberg SL. Fast gapped-read alignment with bowtie 2. *Nat Methods* 2012;9:357–9. 10.1038/nmeth.1923 PMC3322381 22388286

[50] Danecek P, Bonfield JK, Liddle J, et al. Twelve years of SAMtools and BCFtools. *GigaScience* 2021;10:giab008. 10.1093/gigascience/giab008 PMC7931819 33590861

[51] Aroney STN, Newell RJP, Nissen JN, et al. CoverM: read alignment statistics for metagenomics. *Bioinformatics (Oxford, England)* 2025;41:btaf147. 10.1093/bioinformatics/btaf147 PMC11993303 40193404

[52] Camargo AP, Roux S, Schulz F, et al. Identification of mobile genetic elements with geNomad. *Nat Biotechnol* 2024;42:1303–12. 10.1038/s41587-023-01953-y PMC11324519 37735266

[53] Shang J, Cheng P, Liao H, et al. PhaBOX: a web server for identifying and characterizing phage contigs in metagenomic data. *Bioinform Adv* 2023;3:vbad101. 10.1093/bioadv/vbad101 PMC10460485 37641717

[54] Juhász J, Babett B, Juhász J, et al. ProkBERT PhaStyle: accurate phage lifestyle prediction with pretrained genomic language models. *Bioinform Adv* 2025;5:vbaf188. 10.1093/bioadv/vbaf188 PMC12603353 41230491

[55] Buchfink B, Reuter K, Drost H-G. Sensitive protein alignments at tree-of-life scale using DIAMOND. *Nat Methods* 2021;18:366–8. 10.1038/s41592-021-01101-x PMC8026399 33828273

[56] Shang J, Tang X, Sun Y. PhaTYP: predicting the lifestyle for bacteriophages using BERT. *Brief Bioinform* 2023;24:bbac487. 10.1093/bib/bbac487 PMC9851330 36659812

[57] Galperin MY, Alvarez RV, Karamycheva S, et al. COG database update 2024. *Nucleic Acids Res* 2025;53:D356–63. 10.1093/nar/gkae983 PMC11701660 39494517

[58] Hyatt D, Chen G-L, Locascio PF, et al. Prodigal: prokaryotic gene recognition and translation initiation site identification. *BMC bioinformatics* 2010;11:119. 10.1186/1471-2105-11-119 PMC2848648 20211023

[59] Olm MR, Crits-Christoph A, Bouma-Gregson K, et al. inStrain profiles population microdiversity from metagenomic data and sensitively detects shared microbial strains. *Nat Biotechnol* 2021;39:727–36. 10.1038/s41587-020-00797-0 PMC9223867 33462508

[60] R Core Team . R: a Language and Environment for Statistical Computing. Vienna, Austria: R Foundation for Statistical Computing, 2024, https://www.R-project.org/.

[61] Wickham H, Averick M, Bryan J, et al. Welcome to the tidyverse. *J Open Source Softw* 2019;4:1686. 10.21105/joss.01686

[62] Huerta-Cepas J, Szklarczyk D, Heller D, et al. eggNOG 5.0: a hierarchical, functionally and phylogenetically annotated orthology resource based on 5090 organisms and 2502 viruses. *Nucleic Acids Res* 2019;47:D309–14. 10.1093/nar/gky1085 PMC6324079 30418610

[63] Cantalapiedra CP, Hernández-Plaza A, Letunic I, et al. eggNOG-mapper v2: functional annotation, Orthology assignments, and domain prediction at the metagenomic scale. *Mol Biol Evol* 2021;38:5825–9. 10.1093/molbev/msab293 PMC8662613 34597405

[64] Mölder F, Jablonski KP, Letcher B, et al. Sustainable data analysis with Snakemake. *F1000Research* 2021;10:33. 10.12688/f1000research.29032.2 PMC8114187 34035898

[65] Xu Q, Luo G, Guo J, et al. Microbial generalist or specialist: intraspecific variation and dormancy potential matter. *Mol Ecol* 2022;31:161–73. 10.1111/mec.16217 34626522

[66] Lv Y, Fan X, Fan X, et al. Warming increases viral diversity and the relative abundance of virulent viruses in deep soils. *Soil Biol Biochem* 2026;216:110128. 10.1016/j.soilbio.2026.110128

[67] Kosmopoulos JC, Klier KM, Langwig MV, et al. Viromes vs. mixed community metagenomes: choice of method dictates interpretation of viral community ecology. *Microbiome* 2024;12:195. 10.1186/s40168-024-01905-x PMC11460016 39375774

[68] Sorensen JW, Zinke LA, Ter Horst AM, et al. DNase treatment improves viral enrichment in agricultural soil Viromes. *mSystems* 2021;6:e0061421. 10.1128/mSystems.00614-21 PMC8547471 34491084

[69] McDougall WM, Perreira JM, Reynolds EC, et al. CRISPR genetic screens to discover host-virus interactions. *Curr Opin Virol* 2018;29:87–100. 10.1016/j.coviro.2018.03.007 29684735

[70] Lai S, Wang H, Bork P, et al. Long-read sequencing reveals extensive gut phageome structural variations driven by genetic exchange with bacterial hosts. *Sci Adv* 2024;10:eadn3316. 10.1126/sciadv.adn3316 PMC11323893 39141729

[71] Maslov S, Sneppen K. Well-temperate phage: optimal bet-hedging against local environmental collapses. *Sci Rep* 2015;5:10523. 10.1038/srep10523 PMC4451807 26035282

[72] Doekes HM, Mulder GA, Hermsen R. Repeated outbreaks drive the evolution of bacteriophage communication. *eLife* 2021;10:e58410. 10.7554/eLife.58410 PMC7935489 33459590

[73] Aframian N, Bendori SO, Kabel S, et al. Dormant phages communicate via arbitrium to control exit from lysogeny. *Nat Microbiol* 2022;7:145–53. 10.1038/s41564-021-01008-5 34887546

[74] Łoś M, Węgrzyn G. Pseudolysogeny. *Adv Virus Res* 2012;82:339–49. 10.1016/B978-0-12-394621-8.00019-4 22420857

[75] Burke MK . How does adaptation sweep through the genome? Insights from long-term selection experiments. *Proc Biol Sci* 2012;279:5029–38. 10.1098/rspb.2012.0799 PMC3497228 22833271

[76] Abedon S.T. (ed.). Bacteriophage ecology: population growth, evolution, and impact of bacterial viruses. In: Advances in Molecular and Cellular Microbiology, Vol. 15. Cambridge; New York: Cambridge University Press, 2008.

[77] De Paepe M, Hutinet G, Son O, et al. Temperate phages acquire DNA from defective prophages by relaxed homologous recombination: the role of Rad52-like recombinases. *PLoS Genet* 2014;10:e1004181. 10.1371/journal.pgen.1004181 PMC3945230 24603854

[78] Zhou Z, Tran PQ, Martin C, et al. Unravelling viral ecology and evolution over 20 years in a freshwater lake. *Nat Microbiol* 2025;10:231–45. 10.1038/s41564-024-01876-7 39753667

[79] Nadeem A, Wahl LM. Prophage as a genetic reservoir: promoting diversity and driving innovation in the host community. *Evolution* 2017;71:2080–9. 10.1111/evo.13287 28590013

[80] Haerter JO, Sneppen K. Spatial structure and Lamarckian adaptation explain extreme genetic diversity at CRISPR locus. *mBio* 2012;3:e00126–12. 10.1128/mBio.00126-12 PMC3413401 22807565

[81] Wang IJ, Bradburd GS. Isolation by environment. *Mol Ecol* 2014;23:5649–62. 10.1111/mec.12938 25256562

