# Supplementary material for: Temperate versus virulent phage lifestyle impacts microdiversity in soil environments
Source: ISME Commun. 2026 Sep 17;6(1):ycag265. doi: 10.1093/ismeco/ycag265 (PMC13653612; doi:10.1093/ismeco/ycag265)
Supplement: Supplementary file 1 [file tdb_phd_chapter_1_supp_full_v3_ycag265.pdf]

Supplementary Information and Figures - Microdiversity is higher in temperate than in virulent bacteriophages from soil environments

Thomas E.P. de Bruijn, Hilje M. Doekes, Anne Kupczok

Re-clustering of vOTUs using standard thresholds

Re-clustering all the 579,642 vOTUs from the 17 virome datasets using a single set of thresholds (95% average nucleotide identity over at least 85% of the aligned fraction) resulted in 576,980 vOTU clusters, of which 574,460 were singletons (99.6%). Of the 2,520 non-singleton clusters, 2,409 contained two vOTUs, 96 contained three vOTUs, 13 contained four vOTUs, and two clusters contained five and eight vOTUs respectively. There was minor clustering over datasets, exclusively in clusters consisting of two vOTUs. Clusters containing more than two vOTUs only showed clustering within datasets. With the vast majority of the 579,642 vOTUs being clustered into singletons, the unclustered dataset was used for further analyses.

Supplemental Figures

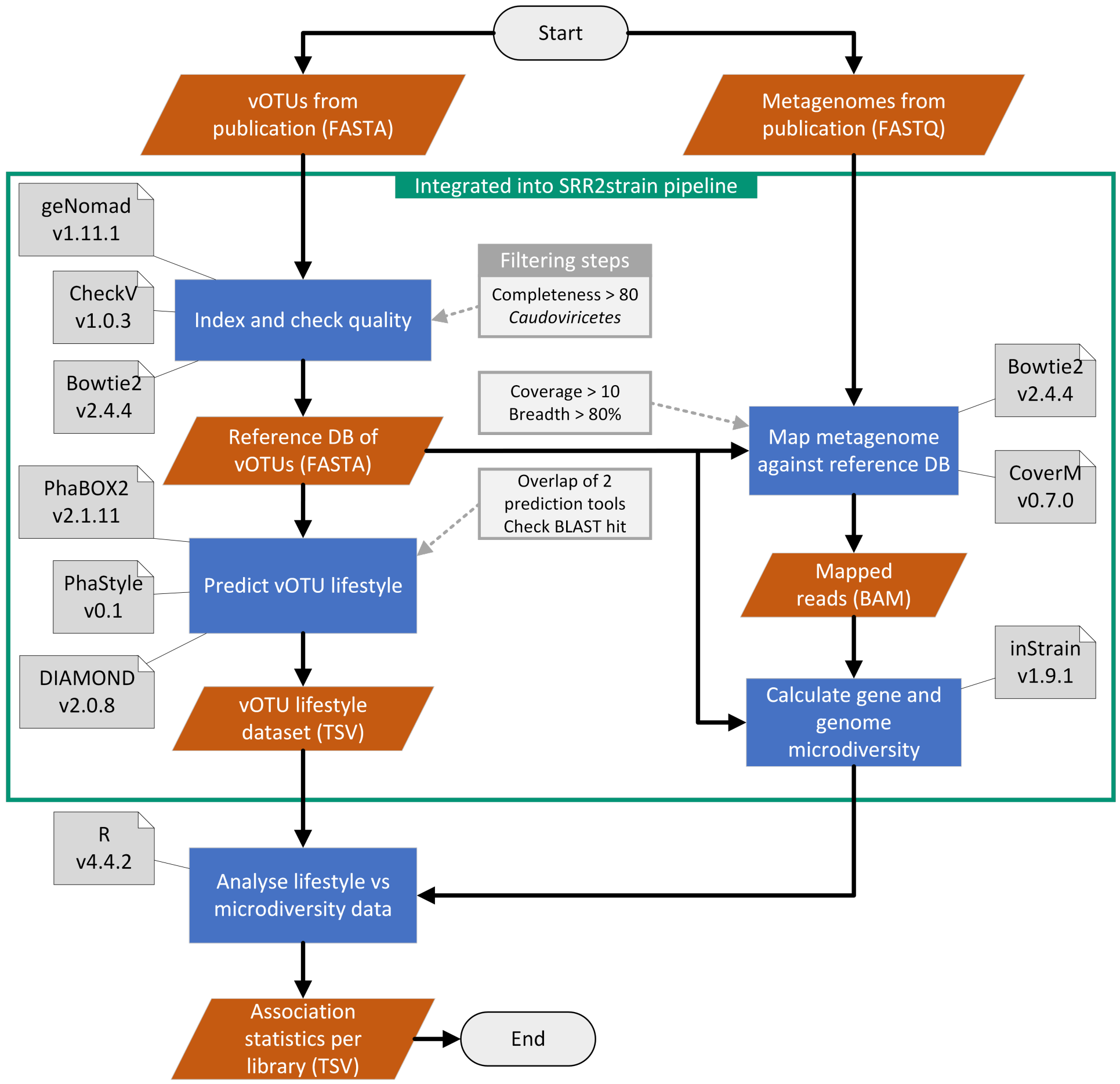

Supplemental Figure 1. Flowchart showing the steps taken and tools used in this study to process raw reads and vOTUs resulting in microdiversity and lifestyle association information. A large part of the steps shown here has been integrated into a snakemake pipeline, enabling high reproducibility and transparency. Version numbers shown next to the tools above can also be found in the Conda environment .yml files. The pipeline is meant to run on a per-dataset basis.

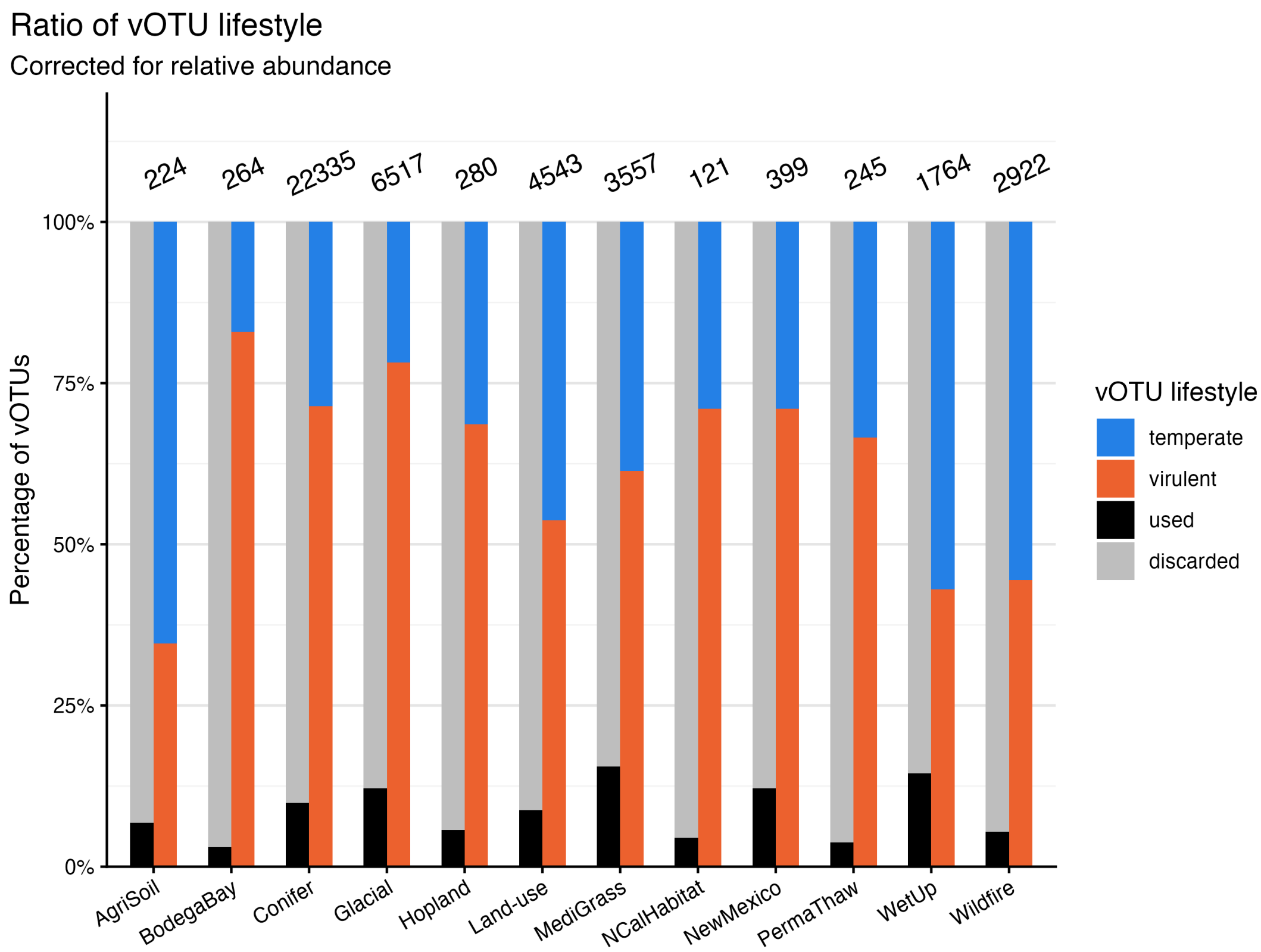

Supplemental Figure 2. Overview of number of vOTUs used in analysis, corrected using relative abundance. Ratios of total and used data, and the ratio between temperate and virulent classified vOTUs. The numbers indicate discarded vOTUs due to quality issues and total vOTUs used for microdiversity analysis. Bar charts used relative abundance as weights, thereby correcting for relative abundance.

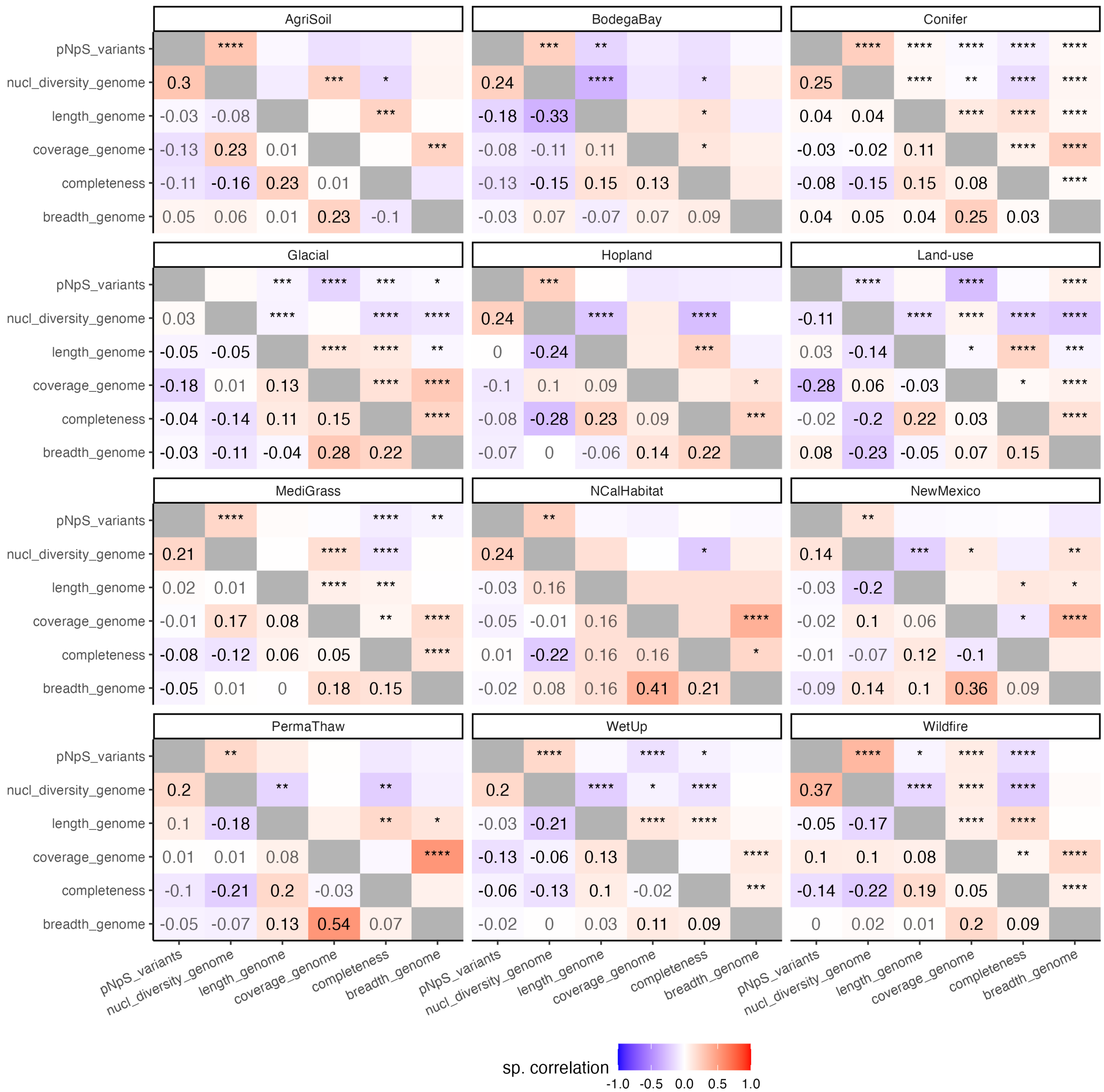

Supplemental Figure 3. Spearman correlations of various quality metrics and nucleotide diversity (i.e. microdiversity) per dataset. Correlations were calculated of the twelve datasets using a Spearman correlation test. Correlation coefficient is shown for p-value < 0.05.

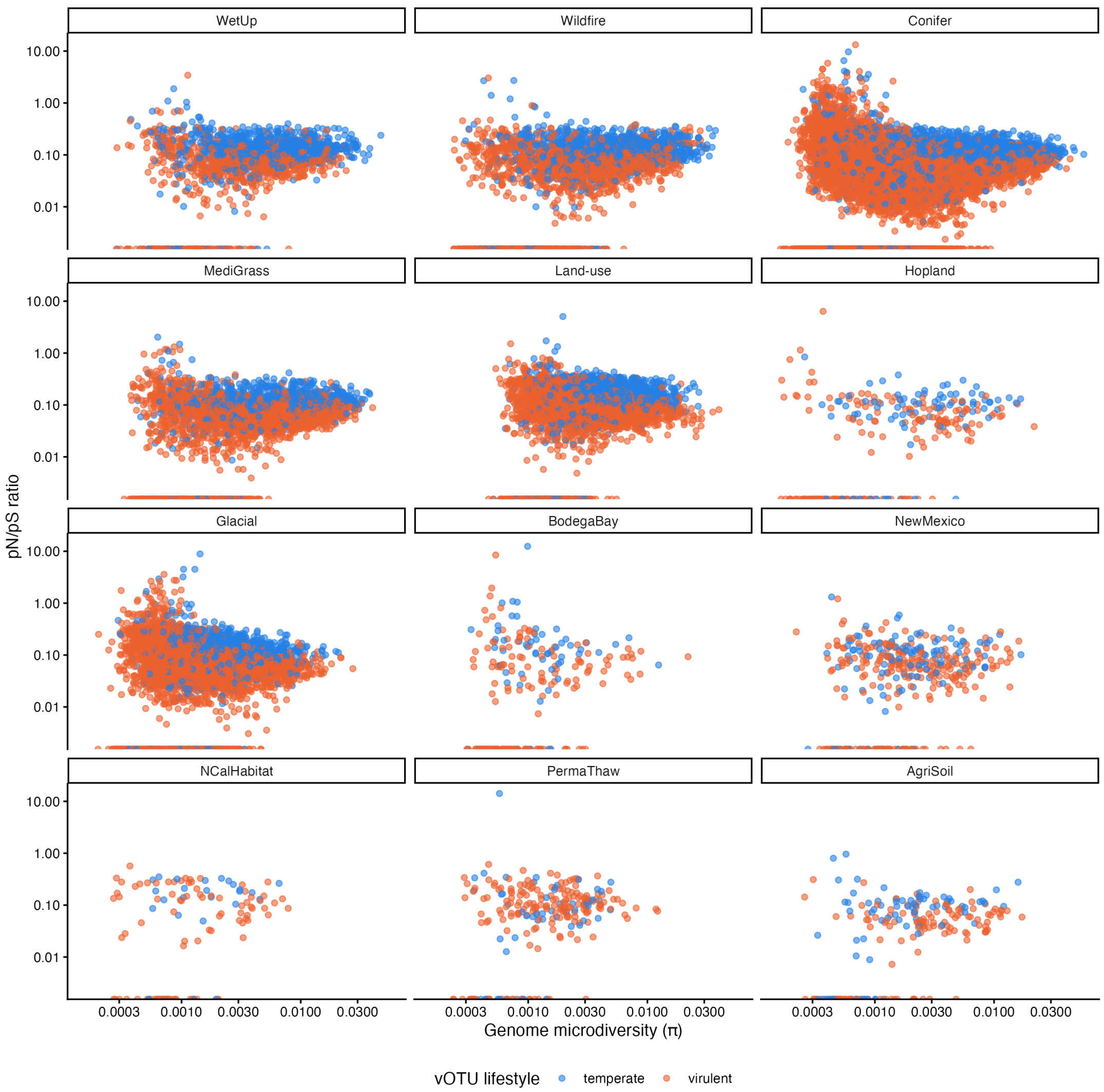

Supplemental Figure 4. pN/pS versus microdiversity plotted per dataset.

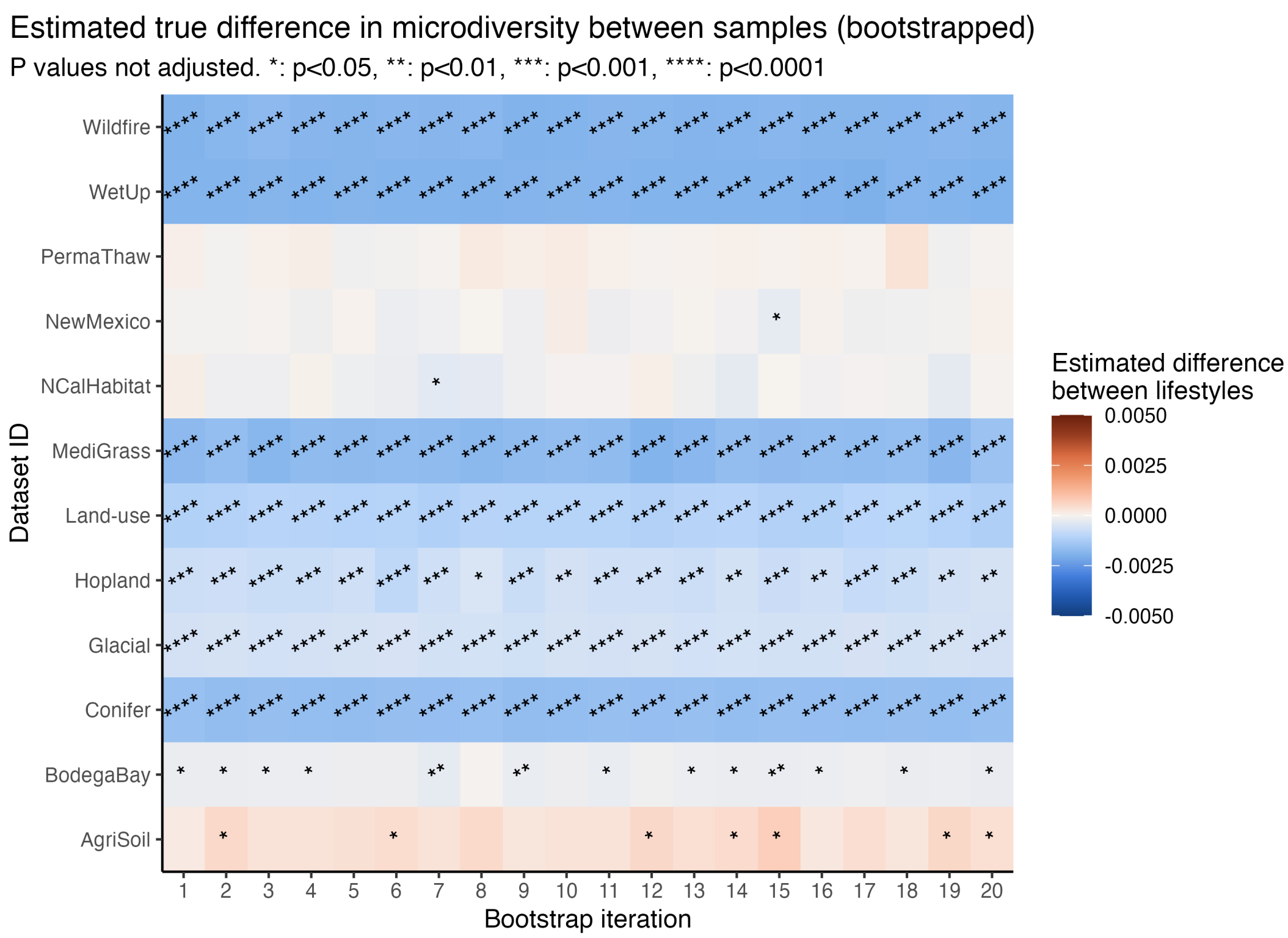

Supplemental Figure 5. Bootstrapping shows robust lifestyle-microdiversity association. Downsampled to lowest abundance lifestyle, Wilcoxon rank sum test for statistical association with asterisks indicating significance. Pattern across bootstrapping iterations is very similar to the non-downsampled results.

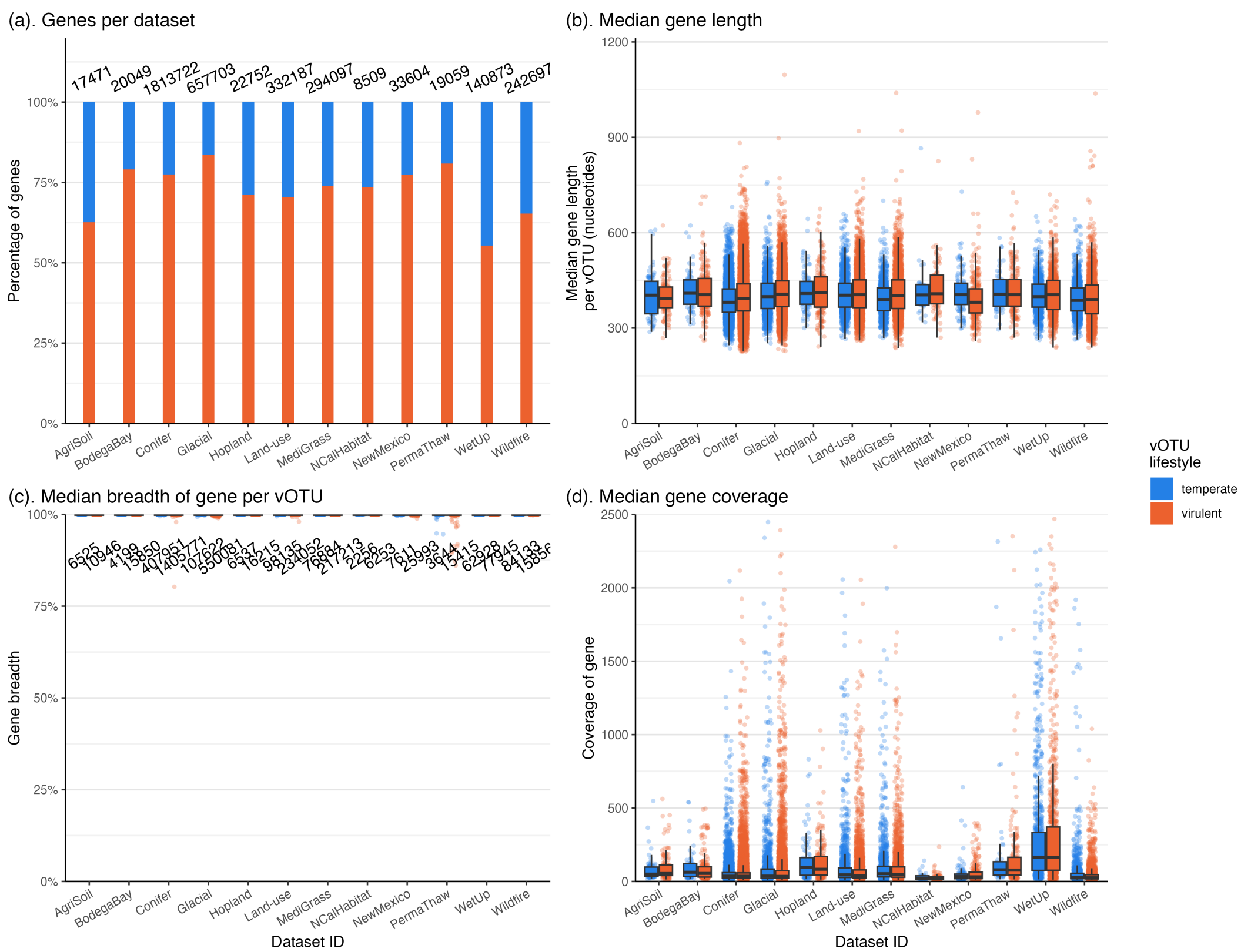

Supplemental Figure 6. General statistics of genes. All genes shown originate from vOTUs that passed the quality thresholds. (**A**). Number of genes per dataset and ratio of lifestyles of the vOTU they originate from. These ratios are similar to the ratios of the vOTUs. (**B**). Median length of genes, split by lifestyle. No patterns are visible due to vOTU lifestyle. (**C**). Median breadth of mapping of genes. These values are higher than those of vOTU breadth. (**D**). Median gene coverage (times). These values are similar to those of vOTU coverage.

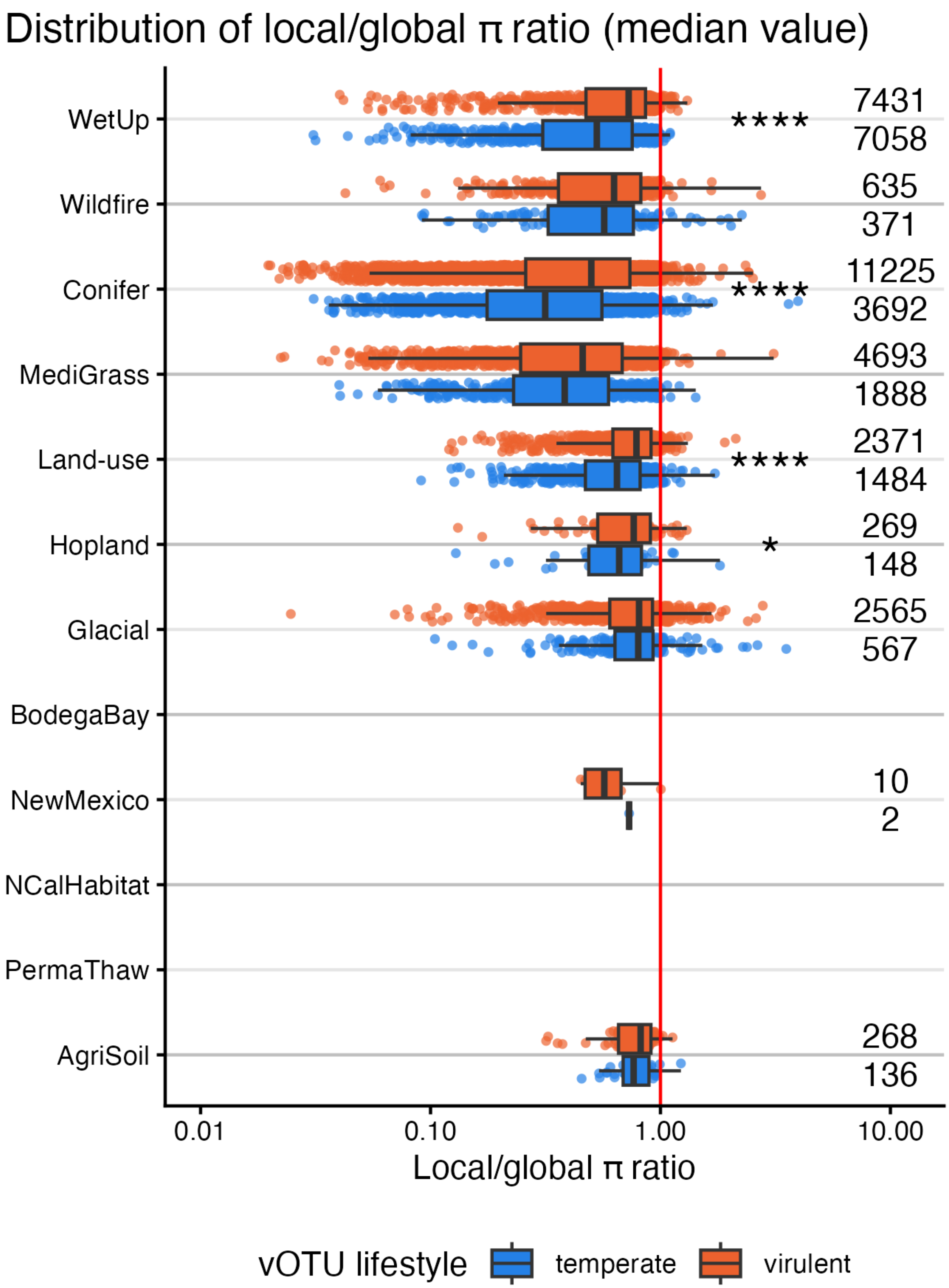

Supplemental Figure 7. Ratio between local versus global diversity per dataset and lifestyle. Temperate phages show generally lower ratio between local and global diversity, implying libraries differ more between each other than within compared to virulent phages.

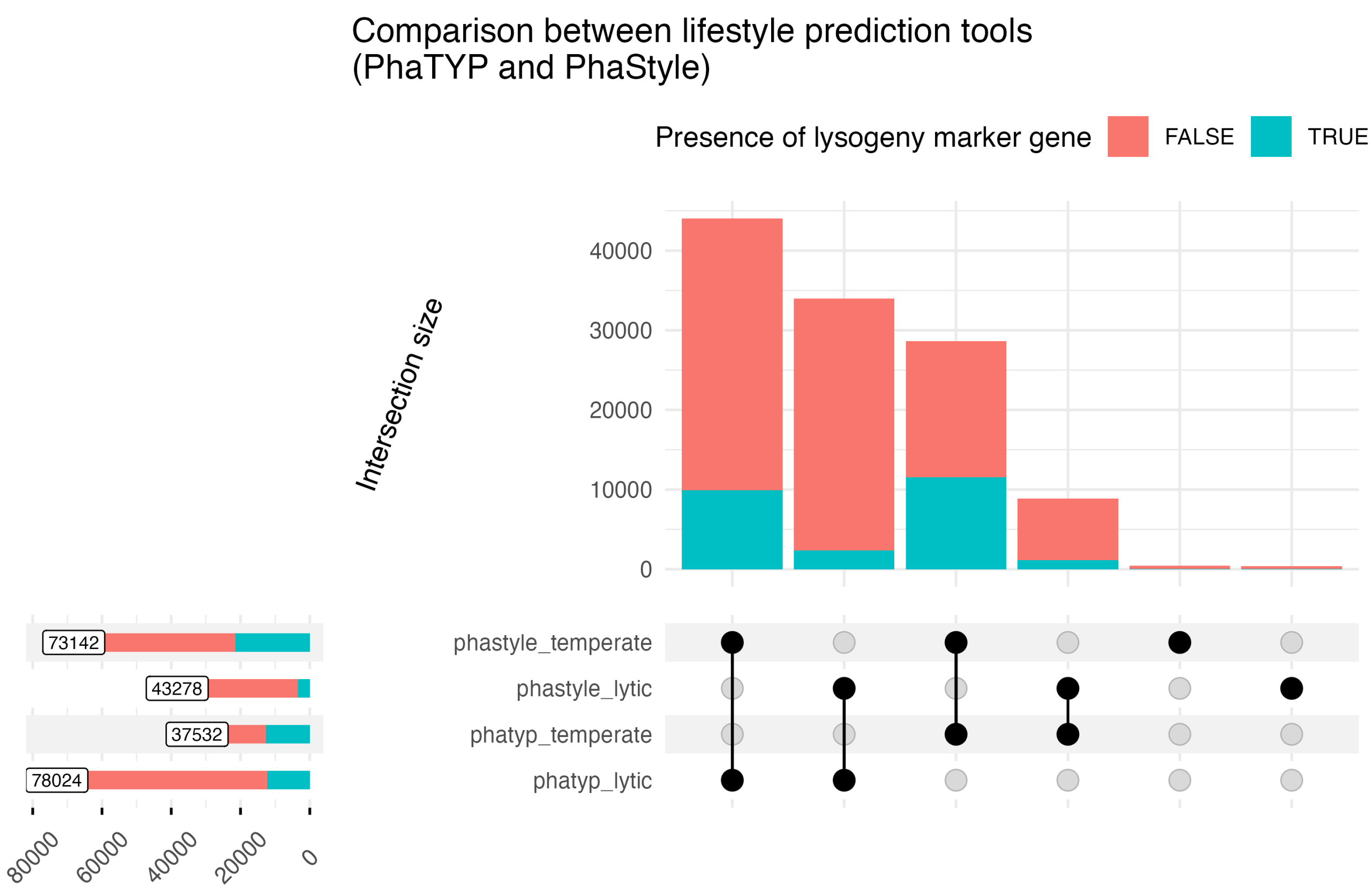

Supplemental Figure 8. Upset plot showing lifestyle prediction groups per tool.

Supplemental Tables

Supplemental Table 1. Mapping efficiencies of all seventeen datasets. The "total reads" column contains the combined number of reads from all included libraries, before mapping. The mapping efficiency, combined with the number of reads, supports the decision to only include viromes in the data analysis, as these tend to have a high mapping efficiency.

Dataset ID	Libraries used for mapping	Mapping efficiency viromes (avg.)	Mapping efficiency metagenomes (avg.)	Total reads
AgriSoil	15 (v) 16 (m)	27.73%	0.42%	518 499 214
BodegaBay	63 (v) 63 (m)	11.30%	2.71%	7 507 979 642
Conifer	90 (v)	64.81%	-	3 371 778 675
Glacial	24 (v)	55.79%	-	1 698 161 279
Hopland	44 (v)	3.24%	-	2 671 824 516
Intertidal	96 (m)	-	0.96%	7 169 436 695
Land-use	50 (v)	37.07%	-	3 168 200 614
MediGrass	59 (v)	30.59%	-	2 777 968 364
NCalHabitat	30 (v)	0.81%	-	1 226 013 651
NewMexico	11 (v)	6.23%	-	983 697 171
PermaThaw	20 (v)	20.66%	-	2 158 529 983
Rhizo	12 (v)	50.18%	-	97 558 067
Spruce	10 (v)	3.28%	-	486 488 059
UKagri	15 (v)	19.78%	-	16 555 479
Watershed	47 (m)	-	1.23%	7 871 535 257
WetUp	152 (v)	21.25%	-	6 487 777 958
Wildfire	39 (v)	48.82%	-	1 452 099 335

Supplemental Table 2. Statistical results regarding association between lifestyle and quality metrics used for filtering. Reference point with regard to estimated true difference is temperate lifestyle (e.g. for length, virulent phages have a longer vOTU sequence). Tested using two-sided Wilcoxon rank sum test.

Metric	Dataset ID	p-value	Estimated true difference
Length	AgriSoil	**5.89e-03**	3 719
Length	BodegaBay	**1.26e-04**	7 012
Length	Conifer	**1.43e-36**	1 947
Length	Glacial	**6.65e-16**	3 113
Length	Hopland	**1.07e-06**	10 038
Length	Land-use	**3.62e-50**	4 516
Length	MediGrass	**2.27e-32**	4 420
Length	NCalHabitat	**4.41e-02**	5 490
Length	NewMexico	**3.65e-10**	11 995
Length	PermaThaw	7.00e-02	-
Length	WetUp	**6.86e-24**	5 101
Length	Wildfire	**7.21e-25**	5 771
Completeness	AgriSoil	**1.98e-02**	0.0000369
Completeness	BodegaBay	2.67e-01	-
Completeness	Conifer	**1.55e-41**	0.0000317
Completeness	Glacial	**4.83e-21**	0.0000938
Completeness	Hopland	3.10e-01	-
Completeness	Land-use	**4.01e-18**	0.0000284
Completeness	MediGrass	**2.28e-04**	0.0000576
Completeness	NCalHabitat	**1.27e-04**	2.50
Completeness	NewMexico	2.38e-01	-
Completeness	PermaThaw	3.70e-01	-
Completeness	WetUp	**6.29e-03**	0.0000477
Completeness	Wildfire	**8.45e-16**	0.0000659
Coverage	AgriSoil	7.99e-1	-
Coverage	BodegaBay	3.74e-1	-
Coverage	Conifer	**2.79e-4**	1.28
Coverage	Glacial	**1.01e-2**	1.96
Coverage	Hopland	9.76e-1	-
Coverage	Land-use	**2.48e-9**	5.76
Coverage	MediGrass	**1.17e-3**	4.60
Coverage	NCalHabitat	9.46e-1	-
Coverage	NewMexico	2.56e-1	-
Coverage	PermaThaw	9.62e-1	-
Coverage	WetUp	9.42e-1	-
Coverage	Wildfire	**1.92e-6**	3.06
Breadth	AgriSoil	8.90e-02	-
Breadth	BodegaBay	7.63e-01	-
Breadth	Conifer	**1.54e-07**	0.0000015
Breadth	Glacial	**3.50e-02**	0.0000023
Breadth	Hopland	4.66e-01	-
Breadth	Land-use	**3.15e-14**	-0.0000106
Breadth	MediGrass	5.79e-02	-
Breadth	NCalHabitat	4.91e-01	-
Breadth	NewMexico	4.08e-01	-
Breadth	PermaThaw	8.60e-01	-
Breadth	WetUp	3.74e-01	-
Breadth	Wildfire	**2.16e-03**	-0.0000017

Supplemental Table 3. General genome statistics per dataset. * The metrics "Length", "Coverage", "Mapping breadth", "Completeness" and "Nucleotide diversity" are all averages per dataset.

Dataset ID	# of vOTUs	Length*	Coverage*	Mapping breadth*	Completeness*	Nucleotide diversity*
AgriSoil	224	50 660	168	1.00	97.6	0.0029
BodegaBay	264	52 060	154	1.00	96.4	0.0014
Conifer	22 335	55 213	61	1.00	98.1	0.0037
Glacial	6 517	68 704	128	1.00	97.4	0.0018
Hopland	280	55 364	165	1.00	97.0	0.0026
Land-use	4 543	49 924	121	0.999	96.7	0.0040
MediGrass	3 557	55 730	113	1.00	98.1	0.0058
NCalHabitat	121	50 126	30	1.00	96.3	0.0017
NewMexico	399	57 154	63	0.999	94.8	0.0026
PermaThaw	245	55 955	412	0.987	95.3	0.0018
WetUp	1 764	53 212	454	1.00	97.6	0.0054
Wildfire	2 922	55 066	62	1.00	97.0	0.0045

Supplemental Table 4. vOTU length and nucleotide diversity statistics per dataset per lifestyle. Clear differences in the number of vOTUs that pass quality thresholds are visible between studies. * The metrics "Length" and "Nucleotide diversity" are averages per dataset.

Dataset ID	Lifestyle	# of vOTUs	Length*	Min. length	Max. length	Nucleotide diversity*
AgriSoil	temperate	85	48 485	21 170	259 025	0.0023
AgriSoil	virulent	139	51 990	13 770	153 522	0.0032
BodegaBay	temperate	65	42 813	14 015	100 963	0.0015
BodegaBay	virulent	199	55 080	13 723	195 821	0.0014
Conifer	temperate	5 281	50 144	14 362	272 124	0.0059
Conifer	virulent	17 054	56 782	12 247	462 798	0.0030
Glacial	temperate	1 381	51 397	13 289	198 996	0.0024
Glacial	virulent	5 136	73 358	12 835	676 544	0.0016
Hopland	temperate	94	45 115	12 996	89 616	0.0033
Hopland	virulent	186	60 543	12 966	316 021	0.0022
Land-use	temperate	1 440	44 774	13 024	164 636	0.0048
Land-use	virulent	3 103	52 314	10 069	373 888	0.0036
MediGrass	temperate	1 003	49 228	13 233	150 206	0.0077
MediGrass	virulent	2 554	58 284	12 793	438 185	0.0050
NCalHabitat	temperate	32	46 905	20 121	95 220	0.0017
NCalHabitat	virulent	89	51 284	15 235	103 915	0.0017
NewMexico	temperate	119	42 863	13 699	88 220	0.0026
NewMexico	virulent	280	63 228	13 967	362 520	0.0027
PermaThaw	temperate	53	48 014	31 941	92 431	0.0017
PermaThaw	virulent	192	58 148	17 908	288 904	0.0018
WetUp	temperate	862	47 423	17 026	144 690	0.0069
WetUp	virulent	902	58 745	13 525	335 613	0.0039
Wildfire	temperate	1 109	48 664	12 788	147 325	0.0063
Wildfire	virulent	1 813	58 982	11 813	357 464	0.0034

Supplemental Table 5. Statistical results regarding association between lifestyle and microdiversity, tested using multiple lifestyle prediction methods. Reference point is temperate lifestyle. Default settings include using PhaTYP, PhaStyle and DIAMOND BLASTp. P-values below 0.05 have been marked in bold. 95% confidence intervals that do not span zero and belong to a p-value below 0.05 are also marked in bold.

Lifestyle tools	Dataset ID	f-statistic	p-value	95% CI low	95% CI high
Default	AgriSoil	4 902	**3.27e-002**	**-0.001021**	**-0.000052**
Default	BodegaBay	7 878	**8.34e-003**	**0.000036**	**0.000367**
Default	Conifer	61 776 988	**0**	**0.001532**	**0.001736**
Default	Glacial	4 939 695	**1.36e-111**	**0.000539**	**0.000648**
Default	Hopland	11 287	**6.99e-005**	**0.000307**	**0.001173**
Default	Land-use	2 917 652	**5.32e-062**	**0.000963**	**0.001206**
Default	MediGrass	1 633 009	**2.16e-037**	**0.001441**	**0.002050**
Default	NCalHabitat	1 504	6.40e-001	-0.000249	0.000431
Default	NewMexico	17 462	4.47e-001	-0.000181	0.000372
Default	PermaThaw	4 874	6.40e-001	-0.000348	0.000230
Default	WetUp	522 570	**6.38e-036**	**0.001589**	**0.002279**
Default	Wildfire	1 386 013	**2.53e-066**	**0.001633**	**0.002058**
PhaStyle only	AgriSoil	42 431	**1.00e- 2**	**-0.000605**	**-0.0000722**
PhaStyle only	BodegaBay	100 810	**6.56e- 3**	**0.0000112**	**0.000152**
PhaStyle only	Conifer	477 079 424	**2.03e-273**	**0.000398**	**0.000488**
PhaStyle only	Glacial	34 044 640	**3.04e-116**	**0.000247**	**0.000279**
PhaStyle only	Hopland	86 037	**6.59e- 3**	**0.0000472**	**0.000337**
PhaStyle only	Land-use	25 219 741	**4.04e-108**	**0.000693**	**0.000831**
PhaStyle only	MediGrass	14 687 043	**8.10e- 51**	**0.000800**	**0.00112**
PhaStyle only	NCalHabitat	15 649	6.30e- 1	-0.000121	0.000210
PhaStyle only	NewMexico	156 359	1.28e- 1	-0.0000413	0.000259
PhaStyle only	PermaThaw	54 548	7.59e- 1	-0.000139	0.000161
PhaStyle only	WetUp	2 985 555	**1.92e- 37**	**0.000903**	**0.00129**
PhaStyle only	Wildfire	8 935 581	**4.64e- 69**	**0.000863**	**0.00110**
PhaTYP only	AgriSoil	62 265	5.19e- 1	-0.000114	0.000200
PhaTYP only	BodegaBay	95 758	**4.12e- 2**	**-0.0000119**	**0.000130**
PhaTYP only	Conifer	388 073 918	**4.11e-33**	**0.000114**	**0.000169**
PhaTYP only	Glacial	26 272 742	**3.01e-29**	**0.000101**	**0.000144**
PhaTYP only	Hopland	91 665	**4.44e- 3**	**0.0000670**	**0.000436**
PhaTYP only	Land-use	23 607 107	**1.55e-22**	**0.000286**	**0.000409**
PhaTYP only	MediGrass	12 980 938	**4.14e-10**	**0.000292**	**0.000572**
PhaTYP only	NCalHabitat	14 757	9.58e- 1	-0.000200	0.000164
PhaTYP only	NewMexico	143 217	6.63e- 1	-0.000126	0.000177
PhaTYP only	PermaThaw	53 278	**3.22e- 2**	**-0.00000592**	**0.000331**
PhaTYP only	WetUp	3 220 506	**8.51e- 7**	**0.000224**	**0.000566**
PhaTYP only	Wildfire	9 002 495	**2.21e-26**	**0.000539**	**0.000766**
PhaStyle + PhaTYP	AgriSoil	14 866	5.27e- 2	-0.000699	-0.0000168
PhaStyle + PhaTYP	BodegaBay	27 154	**1.70e- 3**	**0.0000309**	**0.000226**
PhaStyle + PhaTYP	Conifer	152 551 464	**2.89e-210**	**0.000533**	**0.000622**
PhaStyle + PhaTYP	Glacial	11 358 986	**4.19e-102**	**0.000289**	**0.000357**
PhaStyle + PhaTYP	Hopland	27 838	**3.21e- 4**	**0.000153**	**0.000673**
PhaStyle + PhaTYP	Land-use	8 002 181	**2.13e- 93**	**0.000875**	**0.00107**
PhaStyle + PhaTYP	MediGrass	4 326 644	**3.64e- 43**	**0.00107**	**0.00152**
PhaStyle + PhaTYP	NCalHabitat	4 493	7.36e- 1	-0.000187	0.000264
PhaStyle + PhaTYP	NewMexico	44 012	2.05e- 1	-0.0000979	0.000347
PhaStyle + PhaTYP	PermaThaw	17 735	1.20e- 1	-0.0000425	0.000371
PhaStyle + PhaTYP	WetUp	1 020 429	**2.84e- 30**	**0.00106**	**0.00154**
PhaStyle + PhaTYP	Wildfire	3 077 060	**6.53e- 71**	**0.00119**	**0.00157**

Supplemental Table 6. Statistical results regarding difference between pN/pS of genes between temperate and virulent phages, as tested using a two-sided Wilcoxon rank sum test.

Dataset ID	f-statistic	p-value	95% CI low	95% CI high
AgriSoil	4 180 645	**1.40e-25**	0.0154	0.0333
BodegaBay	1 041 598	**8.40e-13**	0.0079	0.0397
Conifer	57 331 369 800	**0**	0.0279	0.0290
Glacial	2 672 129 586	**0**	0.0313	0.0351
Hopland	6 489 458	**6.60e-50**	0.0288	0.0405
Land-use	2 593 954 210	**0**	0.0431	0.0467
MediGrass	2 571 859 234	**0**	0.0397	0.0423
NCalHabitat	656 806	**2.10e- 5**	0.0000125	0.0334
NewMexico	8 798 160	**3.60e-27**	0.0110	0.0276
PermaThaw	2 063 826	2.47e- 1	-0.0000664	0.0000678
WetUp	600 436 849	**0**	0.0454	0.0494
Wildfire	1 499 329 808	**0**	0.0498	0.0531
